# Liver-specific deletion of miR-181ab1 reduces liver tumour progression via upregulation of CBX7

**DOI:** 10.1007/s00018-022-04452-6

**Published:** 2022-07-22

**Authors:** Jinbiao Chen, Yang Zhao, Fan Zhang, Jia Li, Jade A. Boland, Ngan Ching Cheng, Ken Liu, Jessamy C. Tiffen, Patrick Bertolino, David G. Bowen, Andreas Krueger, Leszek Lisowski, Ian E. Alexander, Mathew A. Vadas, Emad El-Omar, Jennifer R. Gamble, Geoffrey W. McCaughan

**Affiliations:** 1 Liver Injury and Cancer Program Centenary Institute and Sydney Medical School, Faculty of Medicine and Health, The University of Sydney, Camperdown, NSW 2050 Australia; 2 Vascular Biology Program Centenary Institute and Sydney Medical School, Faculty of Medicine and Health, The University of Sydney, Camperdown, NSW 2050 Australia; 3 School of Medicine and Holistic Integrative Medicine, Nanjing University of Chinese Medicine, Nanjing, People’s Republic of China; 4 UNSW Microbiome Research Centre, School of Clinical Medicine, UNSW Medicine and Health, St George and Sutherland Clinical Campuses, Kogarah, NSW 2217 Australia; 5 Centre for Motor Neuron Disease, Department of Biomedical Sciences, Faculty of Medicine and Health Sciences, Macquarie University, Sydney, NSW 2109 Australia; 6 Royal Prince Alfred Hospital, Missenden Road, Camperdown, NSW 2050 Australia; 7 Melanoma Epigenetics Lab Centenary Institute and Sydney Medical School, Faculty of Medicine and Health, The University of Sydney, Camperdown, NSW 2050 Australia; 8 Liver Immunology Program Centenary Institute and Sydney Medical School, Faculty of Medicine and Health, The University of Sydney, Camperdown, NSW 2050 Australia; 9 Molecular Immunology, Faculty of Biology and Chemistry, Justus Liebig University Gießen, Schubertstr 81, 35392 Giessen, Germany; 10 Institute for Molecular Medicine, Frankfurt Cancer Institute, Goethe-University, Frankfurt, Germany; 11 Translational Vectorology Research Unit, Children’s Medical Research Institute, The University of Sydney, Westmead, NSW 2145 Australia; 12 Gene Therapy Research Unit, Children’s Medical Research Institute, Faculty of Medicine and Health, The University of Sydney and Sydney Children’s Hospitals Network, Westmead, NSW 2145 Australia; 13 Laboratory of Molecular Oncology and Innovative Therapies, Military Institute of Medicine, Warsaw, Poland

**Keywords:** Primary liver cancer, microRNA, EMT, AAV, Chromobox family protein, Tumour progression, Cyclin E1

## Abstract

MiR-181 expression levels increased in hepatocellular carcinoma (HCC) compared to non-cancerous tissues. MiR-181 has been widely reported as a possible driver of tumourigenesis but also acts as a tumour suppressor. In addition, the miR-181 family regulates the development and function of immune and vascular cells, which play vital roles in the progression of tumours. More complicatedly, many genes have been identified as miR-181 targets to mediate the effects of miR-181. However, the role of miR-181 in the development of primary tumours remains largely unexplored. We aimed to examine the function of miR-181 and its vital mediators in the progression of diethylnitrosamine-induced primary liver cancers in mice. The size of liver tumours was significantly reduced by 90% in global (GKO) or liver-specific (LKO) 181ab1 knockout mice but not in hematopoietic and endothelial lineage-specific knockout mice, compared to WT mice. In addition, the number of tumours was significantly reduced by 50% in GKO mice. Whole-genome RNA-seq analysis and immunohistochemistry showed that epithelial-mesenchymal transition was partially reversed in GKO tumours compared to WT tumours. The expression of CBX7, a confirmed miR-181 target, was up-regulated in GKO compared to WT tumours. Stable CBX7 expression was achieved with an AAV/Transposase Hybrid-Vector System and up-regulated CBX7 expression inhibited liver tumour progression in WT mice. Hepatic CBX7 deletion restored the progression of LKO liver tumours. MiR-181a expression was the lowest and CBX7 expression the highest in iClust2 and 3 subclasses of human HCC compared to iClust1. Gene expression profiles of GKO tumours overlapped with low-proliferative peri-portal-type HCCs. Liver-specific loss of miR-181ab1 inhibited primary liver tumour progression via up-regulating CBX7 expression, but tumour induction requires both hepatic and non-hepatic miR-181. Also, miR-181ab1-deficient liver tumours may resemble low-proliferative periportal-type human HCC.

**Graphical abstract:**

**Figure:**
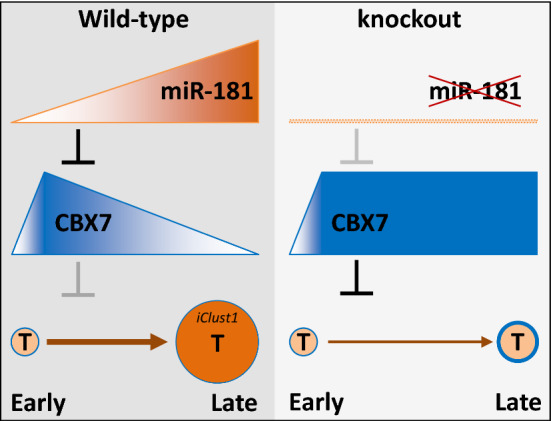
miR-181 was increased with liver tumour growth. More miR-181, darker colour and higher shape. CBX7 was very low in pericentral hepatocytes, increased in early liver tumours, but reduced in advanced liver tumours. Its levels were maintained in miR-181 KO liver tumours. In tumours (T), brown (darker is more) represents miR-181, the blue circle (thicker is more) represents CBX7.

**Supplementary Information:**

The online version contains supplementary material available at 10.1007/s00018-022-04452-6.

## Introduction

Hepatocellular carcinoma (HCC) is the fourth leading cause of cancer-related death worldwide with a steeply rising incidence [1, 2]. Over the past fifteen years, a great effort has been made to find better treatments and classification for HCC patients. Only a few first-line treatments are available for clinical use, such as sorafenib and the combination of atezolizumab and bevacizumab [1, 3]. Meanwhile, multiple molecular classification studies have identified different HCC subtypes, which can guide targeted therapies and improve the prognosis of HCC patients [4–7]. For example, the cancer genome atlas (TCGA) liver cancer study proposed a multi-platform integrative molecular subtyping procedure by which HCC patients could be grouped into three subtypes: iCluster 1, 2, and 3. The iCluster 1 had a significantly worse prognosis than iCluster 2 or 3 [4]. Exploration of different HCC subtypes is desperately needed, including tumour initiation, progression, and therapeutic targets.

Amongst the numerous molecular alterations in HCCs, it is increasingly recognised that microRNAs and epigenetic modifiers play important roles in hepatocarcinogenesis [2, 8–11]. However, their regulatory circuits are very complex [12], and it is crucial to unravel their regulation to fully explore their impact on liver cancer, including its potential treatment in vivo [2, 6, 9, 10, 13–15]. We and others have shown miR-181a induced hepatocyte epithelial–mesenchymal transition (EMT) and was up-regulated in human cirrhosis and HCC [16–19]. More recently, high expression of miR-181a has been shown as one of the features exhibited in iCluster 1 tumours that were associated with a worse prognosis [4]. However, miR-181a modulation could act via either tumour suppression or oncogene induction via different miRNA targets in different cellular compartments [15, 20, 21]. The roles of miR-181 in tumour initiation and progression and its underlying mechanisms need to be further examined with robust approaches [11, 16–19, 22]. Thus, we aimed to investigate the role of miR-181ab1 (Mir181a-1-Mir181b-1 cluster; also called Mirc14), which is the dominant member of the miR-181 family in human HCC (Supplemental data, Figure S1A), in the induction and progression of experimental HCC in vivo. We show for the first time that robust manipulation of miR-181ab1 in vivo using global and tissue-specific knockout mice significantly inhibits primary liver tumour formation and progression. We provide strong evidence that miR-181ab1 promotes tumour progression by inhibiting CBX7, a known miR-181 target and tumour suppressor [17, 23, 24]. We also examined the expression of miR-181 and CBX7 in the TCGA liver cancer database and explored associations with patient outcomes.

## Experimental procedures

All procedures were conducted in accordance with the appropriate ethics and/or institutional review committees. Chemicals, antibodies, and some experimental procedures were described in supplemental data (Supplemental experimental procedures).

### Animals

Mice were housed in a temperature-controlled pathogen-free environment on a cycle of 12-h light and 12-h dark with ad libitum access to food and water. Data obtained from animals were reported according to the ARRIVE guidelines [25].

MiR-181ab1 global knockout mice (referred to herein as GKO) were provided by Prof Andreas Krueger [21]. MiR-181ab1 flox mice (181ab1^f/f^, stock No: 025872), Alb1-Cre mice (stock No: 016832), and R26CreER^T^ (stock No: 004847) were originally from the Jackson Laboratory. VavCre mice were originally from The Walter and Eliza Hall Institute of Medical Research. CBX7 flox (CBX7^f/f^) mice were obtained from Dr Haruiko Koseki through the Japan RIEN BRC [26, 27]. Backcrossed miR-181ab1^f/f^ mice were crossed with Alb1-Cre mice to generate miR-181ab1^f/f^:Alb1-Cre^+^ (i.e. hepatocyte-specific KO, referred to herein as LKO), with R26CreER^T^ mice to generate miR-181ab1^f/f^:R26CreER^T^ (i.e. inducible KO, referred to herein as iKO), and with vav-Cre mice to generate miR-181ab1^f/f^:vav-Cre^+^ (i.e. hematopoietic and endothelial cells-specific KO, referred to herein as vavKO) on the C57BL/6J background. 181ab1^f/f^ and CBX7^f/f^ mice were crossed with Alb1-Cre mice to generate miR-181a/b-1^f/f^:CBX7^f/f^:Alb1-Cre^+^ (i.e. hepatocyte-specific miR-181ab1 and CBX7 double knockout, referred to herein as DKO). Control mice (referred to herein as WT) were corresponding Cre negative flox littermates or Cre only littermates of the above mice. Genotyping was performed as described before (Supplemental experimental procedures) [28, 29]. Primary liver tumours were induced with diethylnitrosamine (DEN) in mice as described before [29–32].

### RNA-seq analysis

Preparation of cDNA libraries and high‐throughput sequencing (conducted on the Illumina HiSeq platform) were performed by Novogene (Beijing, China). After quality control procedures, short reads were mapped to mouse genome assembly (mm10) by STAR version 2.7.3a [33]. Mapped reads were quantified by featureCounts version 2.0.0 from the Subread package [34]. Ward’s hierarchical clustering method was applied to group the samples based on the gene expression matrix. Differential gene expression analysis was then conducted using the R package Limma. Gene set enrichment analysis was subsequently performed with the R package clusterProfiler [35]^.^ Overlaps between differentially expressed gene sets and gene sets in MSigDB were computed with Gene Set Enrichment Analysis (GSEA) [36].

### Primary mouse liver tumour cells

The culture of primary mouse liver tumour cells was described previously [37]. In brief, tumour tissues were minced, washed with DPBS, and then incubated with type IV collagenase (0.05% in Hank’s BSS with Ca^2+^, pH 7.4; C5138, Sigma–Aldrich) at 37 °C for up to half hour. Cells were dissociated by pipetting, filtered through a 70 μm cell strainer, and pelleted by centrifugation at 50*g* for 4 min. Cell pellets were washed once with DPBS, re-suspended with medium (DMEM/F12 with 20% heat inactivated foetal bovine serum, 1% l-glutamine, 1% penicillin–streptomycin, 0.01 g/L hydrocortisone hemisuccinate, 0.01 g/L insulin and 20 μg/L EGF) and plated in several wells of 6-well plates (termed as passage 0). Upon confluent, cells were trypsinised and passaged for experiments. All cells were cultured at 37 °C with 5% CO_2_ and 95% air. In the tamoxifen (TAM)-induced knockout assay, 100 nM 4-OHT medium was added and incubated for 3 days as previously describe [28].

### The hybrid recombinant adeno-associated viral vector (rAAV)‐piggyBac gene therapy system

Recombinant adeno-associated viral (rAAV) vectors have been widely used for liver-targeted gene transfer in preclinical models. Conventionally, rAAV vectors do not form somatic integrations. Thus, proliferating cells, such as tumour cells, would divide and rapidly dilute out conventional rAAV vectors. A novel hybrid rAAV‐piggyBac gene therapy system was used to achieve stable gene expression in hepatocytes and liver tumour cells [16, 38]. In brief, a new rAAV construct named pAAV2‐TRsh.LP1a was created that carries short piggyBac terminal repeats, which can efficiently mediate gene transposition into mammalian cells in vivo in combination with piggyBac transposase (transposase-AAV) [38]. CBX7 was taken from pTRIPZ (M)-HT-Cbx7, which was a gift from Xiaojun Ren (Addgene plasmid # 82515; http://n2t.net/addgene:82515; RRID: Addgene_82515), and cloned into pAAV2‐TRsh.LP1a to form a new vector, named as pAAV2‐TRsh.LP1a-CBX7 (CBX7-AAV). Its control vector was pAAV2‐TRsh.LP1a-GFP (GFP-AAV). All rAAV were pseudo‐serotyped with the AAV8 capsid and produced in HEK293 cells as described previously [38]. Single doses of 1E + 11vgc of both CBX7-AAV and GFP-AAV with transposase-AAV were given by intravenous injection to mice at week 20 post-DEN injection. AAV-treated mice were harvested at week 14 post-AAV injection.

### TCGA LIHC data and survival analysis

TCGA liver cancer data were downloaded either from OncoLnc or cBioPortal [39]. Kaplan–Meier survival analysis was conducted with the Prism software.

### Biostatistical analysis

Data were expressed as mean ± SEM. Data transformation was conducted if needed by biostatistical analysis. Analysis of variance (ANOVA), Mann–Whitney test, Kruskal–Wallis test, and Student’s *t* test, were used to analyse the differences amongst groups and a p value less than 0.05 was regarded statistically significant.

## Results

### Global and hepatocyte-specific deletion of miR-181ab1 inhibits liver tumour initiation and progression

We have previous showed that miR-181a induced hepatocyte EMT in vitro and was over-expressed in both human and mouse HCC and cirrhotic livers [16], suggesting that miR-181 might be an important driver of liver cancer. To explore whether miR-181 inhibition could block the development of liver cancer, we examined the development of primary liver cancer in mice by knocking out the miR-181ab1 cluster, which accounts for most mature miR-181 in human HCC (Supplementary data, Fig. S1A), and is highly conserved across human and mouse species [18].

**Fig. 1:**
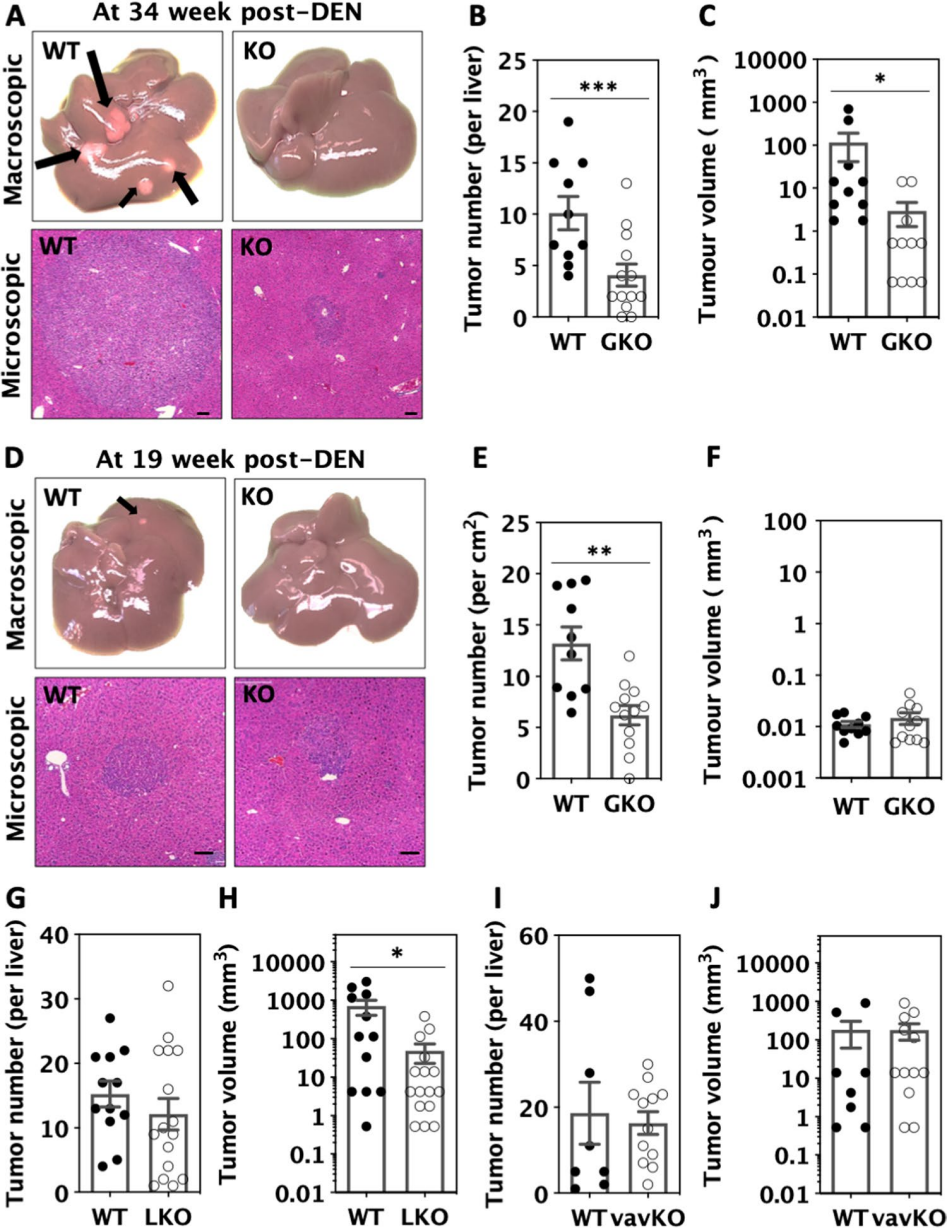
Effects of miR-181ab1 deletion on liver cancer formation. DEN was injected intraperitoneally once into miR-181ab1^GKO^ (GKO), miR-181ab1^LKO^ (LKO), miR-181ab1^vavKO^ (vavKO) and control WT male pups at postnatal day 12 ~ 14. Liver tissues were harvested at 34-week (**A**–**C**, **G**–**J**) and 19-week (**D**–**F**) post-DEN injection. Visible tumours on the surface of each liver were counted and their size measured. **A** and **D** Graphs of liver tumours, **B**, **E**, **G** and **I** Liver tumour number, **C**, **F**, **H** and **J** Liver tumour volume. WT and KO data were compared with unpaired t test after data transformation if needed in Prism, *p < 0.05, **p < 0.01, ***p < 0.001

We found that the number of tumours in GKO livers was reduced by 50% and the tumour volume by 90% compared to WT livers at 34-week post-DEN injection (Fig. 1A–C). At week 19 only tumour number was reduced, indicating that global miR-181 may regulate tumour initiation (Fig. 1D–F). As GKO mice had previously been shown to have an altered immune phenotype, we established hepatocyte-specific (LKO) and hematopoietic/endothelial specific miR-181ab1 knockout mice (vavKO). Tumour numbers at week 34 were similar in LKO livers and vavKO mice compared with WT littermates (Fig. 1G, I). However, the tumour volume in LKO but not in vavKO livers was dramatically and significantly reduced by 90% (similar to GKO livers) compared to WT livers at 34-week post-DEN injection (Fig. 1C, H, J). Thus, our data would suggest that the progression of primary liver tumours was inhibited by miR-181ab1 loss in the hepatic compartment.

### MiR-181ab1 knockout reduces liver cancer cell proliferation in vivo and in vitro

**Fig. 2:**
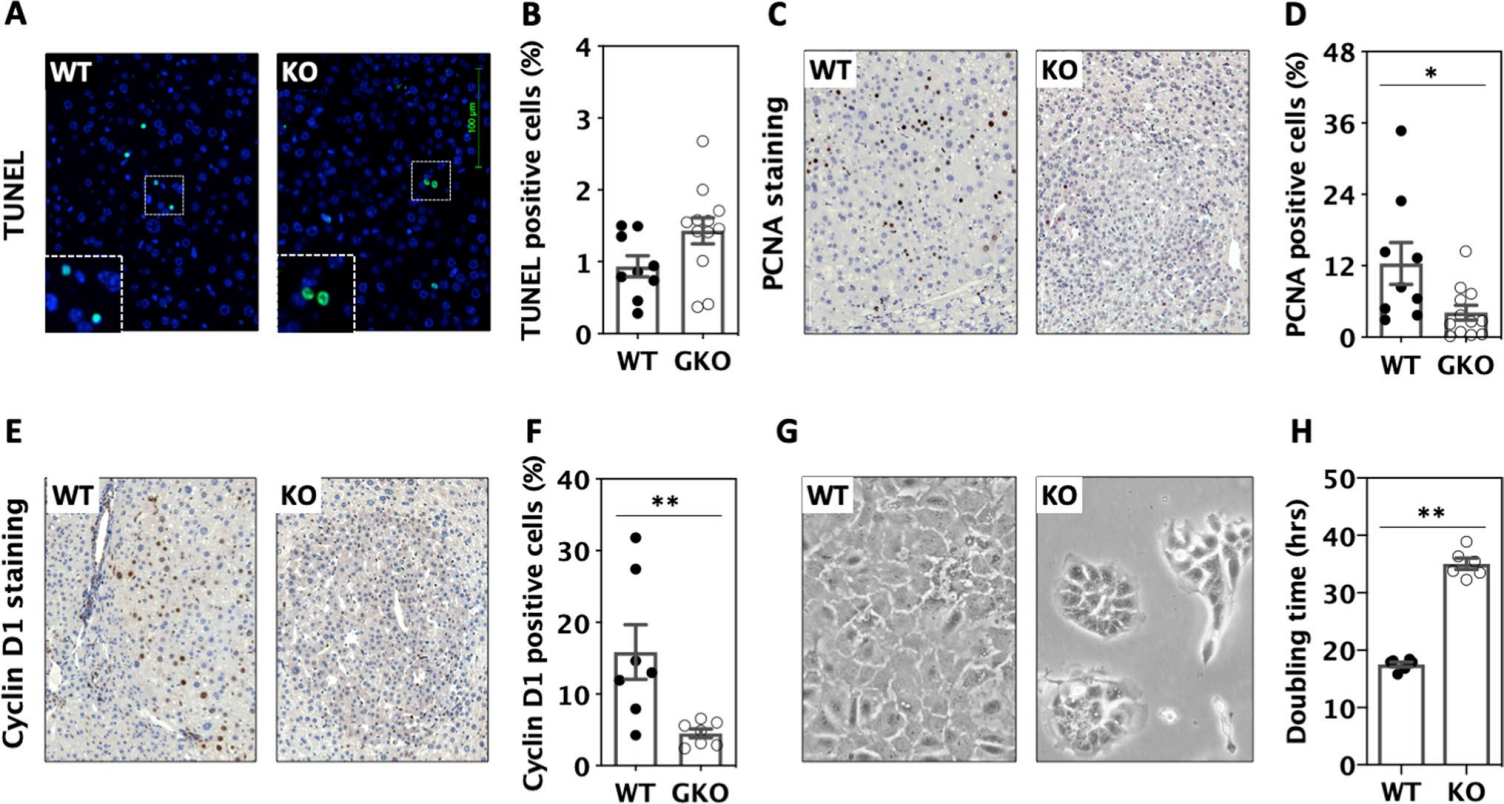
Deletion of miR-181ab1 reduces liver tumour growth. TUNEL, PCNA and cyclin D1 were used to detect apoptotic cells and proliferating cells in tumours harvested from wild type (WT) and global knockout (GKO) mice treated with DEN for 34 weeks (**A**–**F**). **A** and **B** The percentage of TUNEL-positive cells, **C** and **D** The percentage of PCNA-positive cells, and **E** and **F** The percentage of cyclin D1-positive cells. **G** and **H** Primary tumour cells were isolated and established from WT and GKO liver tumours and doubling time of cells was determined from 3 different cell lines at different passages per genotype. Data were analysed by unpaired t test after data transformation if needed in Prism, *p < 0.05, **p < 0.01

To examine the underlying mechanisms responsible for the inhibitory effects of miR-181ab1 deletion on liver tumour progression, we measured apoptosis in liver tumours with TUNEL assays and found that the percentage of apoptotic cells in GKO tumours was similar to that in WT tumours (Fig. 2A and B). In contrast, tumour cell proliferation measured with PCNA and cyclin D1 was significantly reduced in GKO tumours compared with WT tumours in vivo (Fig. 2C–F).

Consistent with a role of miR-181ab1 in inducing tumour proliferation in vivo, isolated and cultured miR-181ab1 KO primary liver tumour cells had significantly longer doubling time compared with WT tumour cells in vitro (Fig. 2G and H). Thus, miR-181ab1 loss inhibited liver tumour progression, at least partially, by reducing tumour cell growth, rather than increasing the apoptosis of tumour cells.

### HCC and cell cycle signatures are differentially enriched in miR-181ab1 deficient liver tumours

**Fig. 3:**
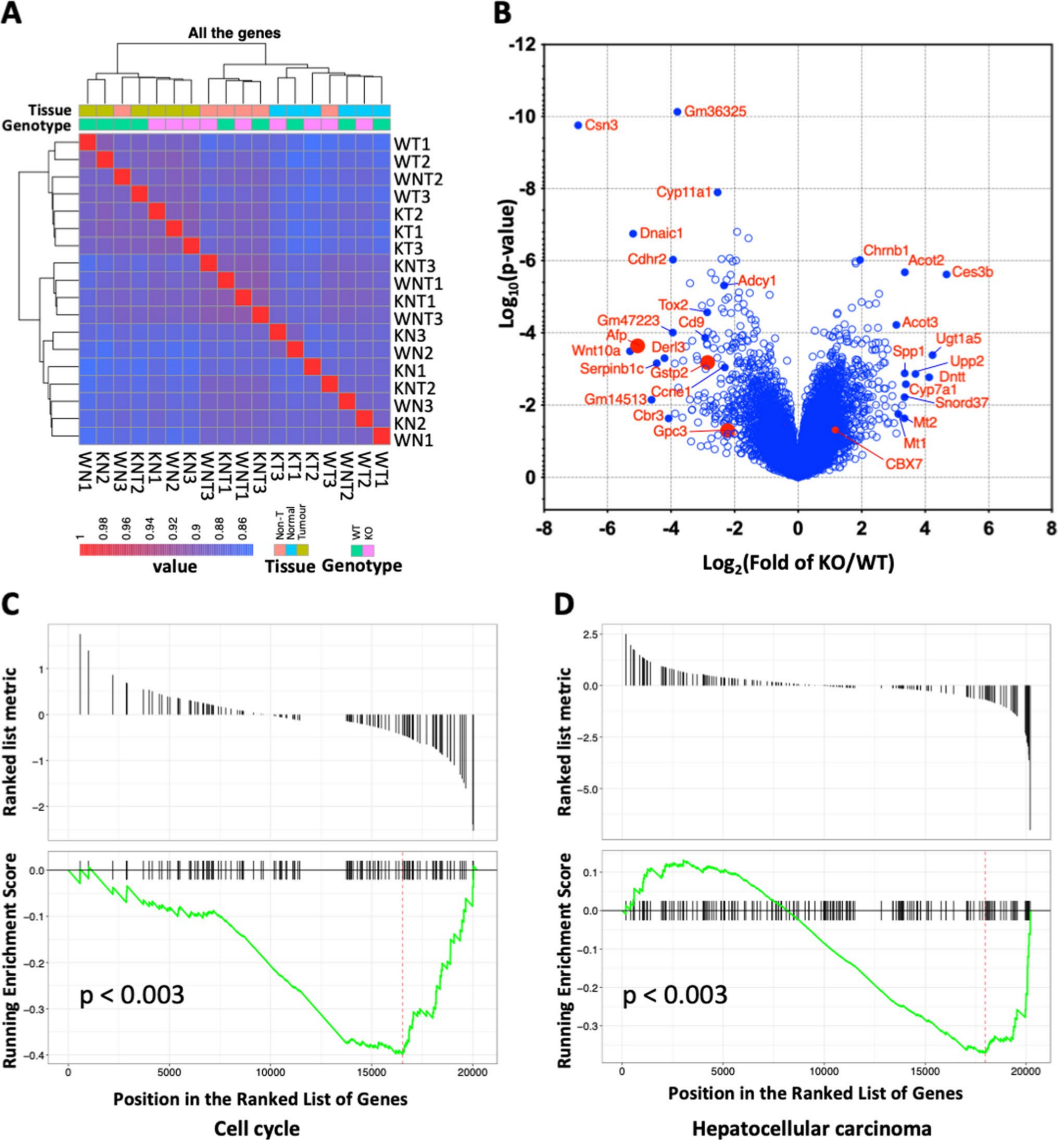
RNA-seq data. Whole genome gene expression profiles were obtained from 18 samples, i.e. WT tumours (WT), WT non-tumours (WNT), KO tumours (KT), KO non-tumours (KNT), WT normal livers (WN) and KO normal livers (KN). **A** The cluster analysis of samples based on expression profiles of all the genes, **B** the volcano analysis of differentially expressed genes (DEG) between KO and WT tumours, Gene Set Enrichment Analysis (GSEA) of KEGG cell cycle (**C**) and hepatocellular carcinoma (**D**) gene sets

To identify the molecular pathways and genes regulated by miR-181ab1 that promoted tumour growth, we conducted RNA-seq to obtain whole-genome gene expression profiles of tumours, non-tumours and normal liver tissues from WT and GKO mice. Unbiased cluster analysis confirmed that tumour, non-tumour, and normal liver samples clustered in distinct groups. WT and GKO tumour samples were also clustered separately (Fig. 3A). Next, we compared the gene expression profiles of WT and GKO tumours and identified *n* = 2424 differentially expressed genes (DEGs). Liver tumour markers, such as AFP, GSTp2, and GPC3, in GKO tumours were reduced in comparison to WT tumours (Fig. 3B). Consistent with the reduced proliferation rate of liver tumour cells in GKO mice (Fig. 2C–H), KEGG_GSEA analysis revealed that expression levels of hepatocellular carcinoma and cell cycle-associated genes were lower in KO than WT tumours (Fig. 3C and D). Computing overlaps with chemical and genetic perturbations (CGP) and hallmark (H) gene sets in the Molecular Signatures Database (MSigDB) revealed that 502 genes were up-regulated in GKO tumours vs WT tumours, with significantly overlapped with genes down-regulated in liver cancer compared to normal liver tissues (Supplemental data, Table S2).

### Deletion of miR-181ab1 increases expression of E-Cadherin

**Fig. 4:**
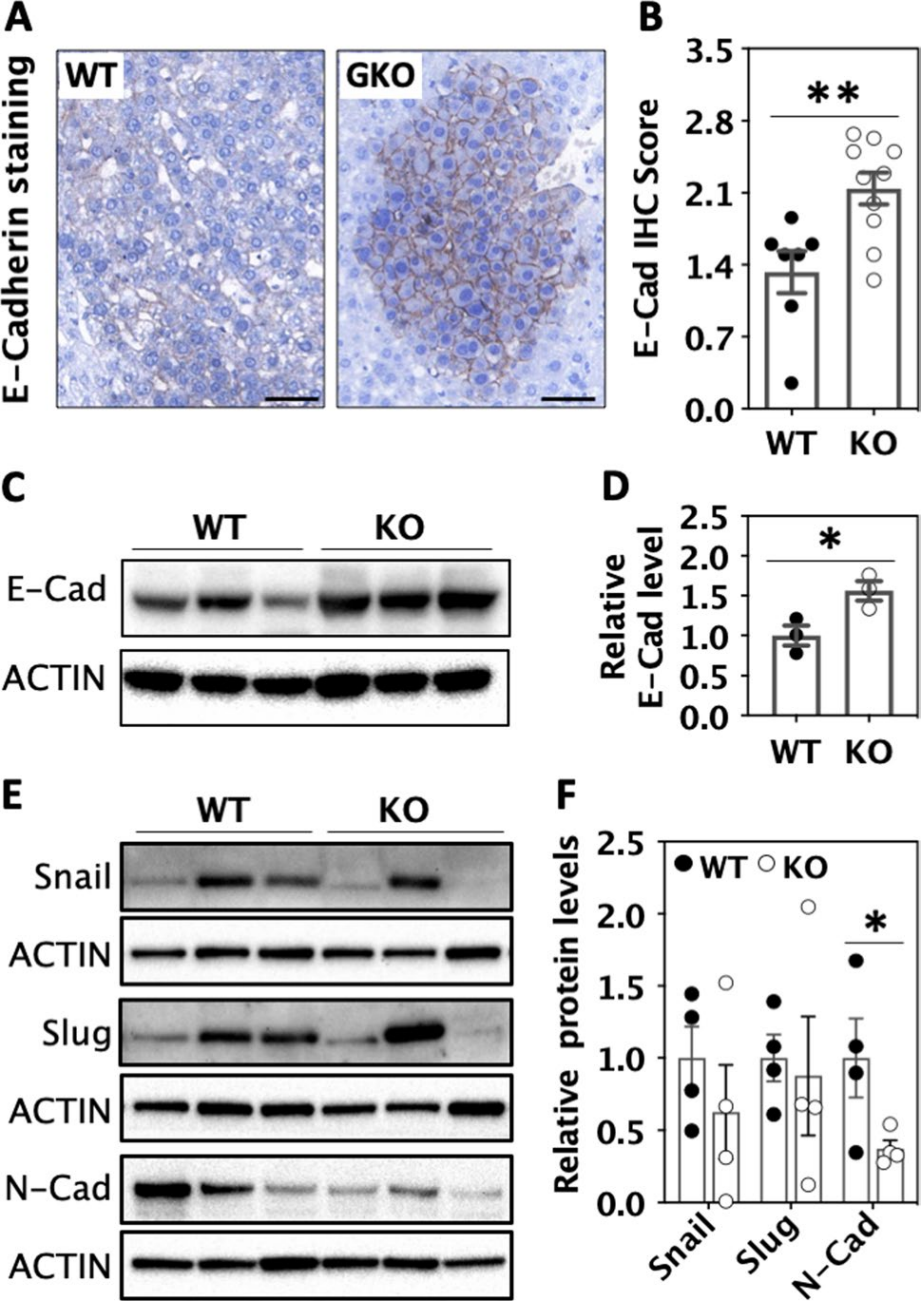
MiR-181ab1 plays a role in EMT. Tumours harvested from wild type (WT) and global knockout (GKO) mice treated with DEN for 34 weeks. **A** Micrographs and **B** quantification of E-cadherin (E-Cad) immuno-histochemical staining. **C** Micrographs and **D** quantification of E-cadherin (E-Cad) Western blotting of primary liver tumour cells. **E** Micrographs and **F** quantification of Snail, Slug, and N-cadherin (N-Cad) Western blotting of primary liver tumour tissues (*n* = 4). Note: Snail and Slug share ACTIN with WNT10a and DUSP4 in Fig. 5 as the same membranes were used. Data were compared with unpaired Student’s *t* test in Prism, *p < 0.05, **p < 0.01

The miR-181 family has been widely reported to mediate TGF-beta signalling and EMT [16, 19, 40]. After comparing DEGs with CGP and H gene sets in MSigDB, we found many DEGs were Smad2/3 targets or EMT hallmark genes, or regulated by TGF-beta superfamily ligands (Table 1). E-Cadherin is a key EMT marker. Its expression is reduced upon EMT. Analysis of E-cadherin in liver tumours by immuno-histochemical staining (Fig. 4A, B) and in isolated liver tumour cells by Western blotting (Fig. 4C, D) showed a significant increase in the protein expression level of E-Cadherin in GKO tumour cells compared with WT tumour cells. The expression of other EMT markers, such as Snail, Slug, and N-Cadherin, in liver tumour tissues was also examined with western blotting (Fig. 4E, F). The results showed that the protein expression levels of N-Cadherin but not Snail and Slug were significantly lower in GKO than WT tumour tissues, suggesting that deletion of miR-181ab1 partially reversed EMT in liver tumours independent of Snail or Slug.

**Table 1:** DEGs overlap with other gene sets in MSigDB

Gene set	Description	Overlap	k/K	FDR *q* value
Genes up-regulated in KO tumours vs WT tumours (502 genes in comparison)				
Hallmark_EMT	Genes defining EMT, as in wound healing, fibrosis and metastasis	31	0.155	3.16E−21
Lee_BMP2_targets_UP	Genes up-regulated in uterus upon knockout of BMP2	80	0.104	8.55E−44
Genes down-regulated in KO tumours vs WT tumours (340 genes in comparison)				
KOINUMA_targets_of_Smad2_or_3	Genes with promoters occupied by SMAD2 or 3 in HaCaT cells according to a ChIP-chip analysis	23	0.027	1.14E−04
ACEVEDLee_BMP2_ targets_DN	Genes down-regulated in uterus upon knockout of BMP2	24	0.027	9.92E−05

### Identification of CBX7 as a strong miR-181 target candidate regulating liver tumour progression

**Fig. 5:**
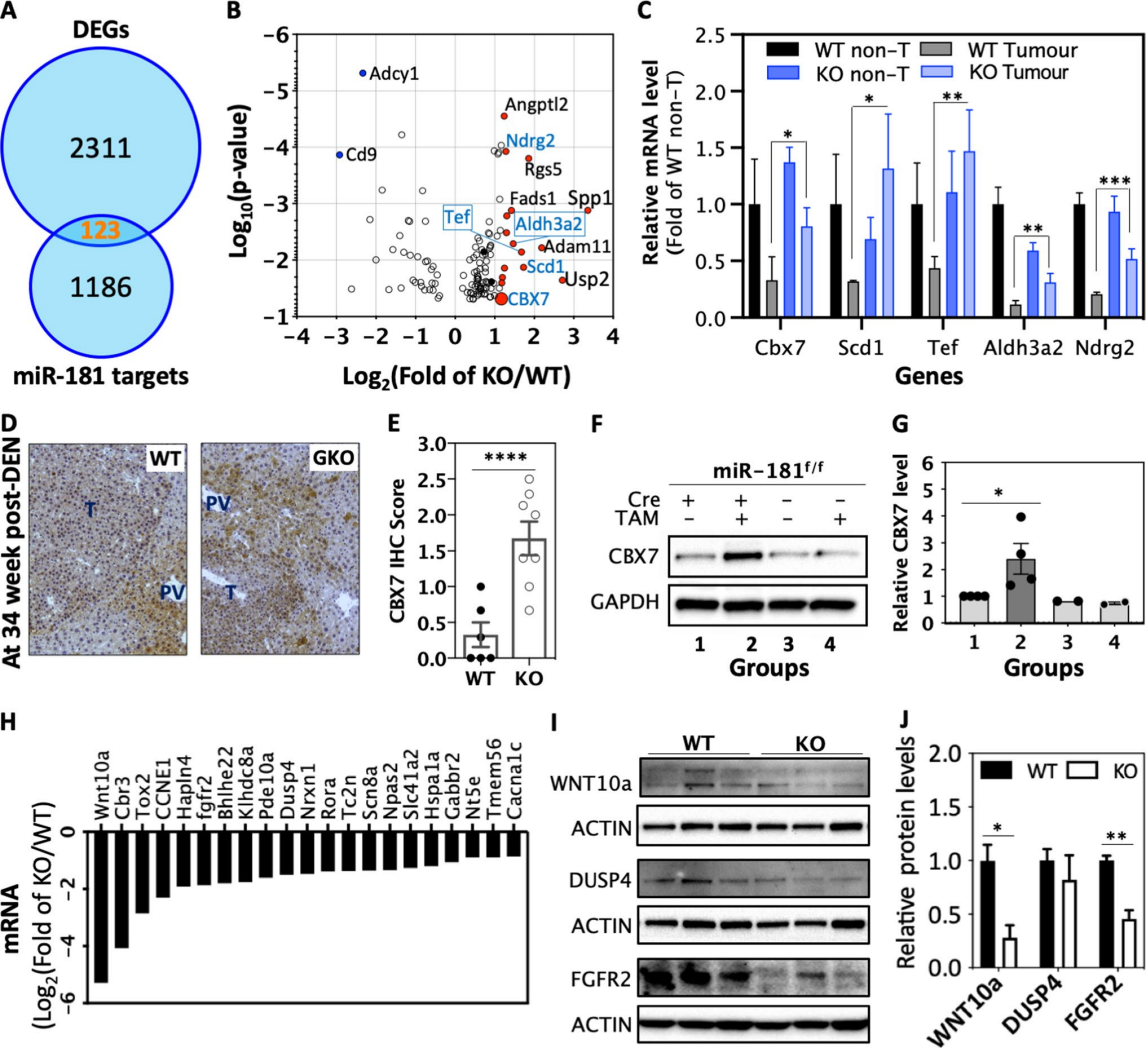
CBX7 is a strong candidate for mediating effects of miR-181 on liver tumour progression. **A** Differential expressed genes (DEGs) in miR-181ab1 WT and KO liver tumours were compared with miR-181 target genes, which were obtained from miRTarbase. **B** Expression levels of 123 overlapped genes were shown in a volcano graph. **C** Five candidates of miR-181 target genes and their expression levels in WT and KO tumour and non-tumour (non-T) tissues. Data were compared with unpaired Student’s *t* test in Prism, *p < 0.05, **p < 0.01, ***p < 0.001. **D** and **E** CBX7 expression in miR-181 WT and global knockout (GKO) tumours harvested from mice treated with DEN for 34 weeks. **F** and **G** Tumour cells isolated from the miR-181 flox (miR-181^f/f^) liver tumours with or without tamoxifen (TAM) inducible Cre were treated with TAM and CBX7 expression was examined with Western blotting assays. *p < 0.05, Kruskal–Wallis test. **H** Top CBX7 target genes in DEGs down-regulated in miR-181 KO tumours compared to WT tumours. **I** and **J** WNT10a, DUSP4 and FGFR2 expression in miR-181 WT and KO liver tumour tissues (*n* = 4). Note: WNT10a and DUSP4 share ACTIN with Snail and Slug in Fig. 4 as the same membranes were used. Data were compared with unpaired Student’s t test in Prism, *p < 0.05, **p < 0.01

In searching for individual genes or pathways that mediate the functions of miR-181 in liver tumour progression, we firstly compared DEGs with known miR-181 targets as listed in the miRTarbase [41]. Then we found that 123 miR-181 target genes were altered in GKO tumours compared with WT tumours (Fig. 5A, B). We presumed key miR-181 targets would be lower in liver tumours than non-tumour tissues in WT mice. MiR-181 target genes would be significantly up-regulated (> twofold) in KO tumours compared to WT tumours (Fig. 5B). We identified 16 genes that met these criteria (Supplemental data, Figure S3A). Then, we compared the expression of these 16 genes in three iClusters of human HCC in which the miR-181 expression was higher in iClust1 than iClust2 or iClust3. We found the expression levels of 5 genes, namely Scd1, Tef, Ndrg2, Aldh3a2, and CBX7, were decreased in iClust1 compared with iClust2 or iClust3 liver tumours (Supplemental data, Figure S3B). Thus, Scd1, Tef, Ndrg2, Aldh3a2, and CBX7 are the most likely candidates for mediating effects of miR-181 on liver tumour progression (Fig. 5C). Scd1 is dispensable for liver cancer formation [42]. Tef and Ndrg2 promote tumour cell survival and metastasis [43, 44]. The expression level of Aldh3a2 is increased in human HCC compared to normal liver tissues according to the Human Protein Atlas and GEPIA [45, 46], but it does not predict human HCC survival [4]. Thus, we focussed on CBX7 as the most likely mediator of miR-181 effects in liver cancer progression.

CBX7 contains two miR-181-binding sites (Supplemental data, Figure S4) and has been independently demonstrated to be a miR-181 target by multiple groups [17, 23, 24]. Furthermore, CBX7 had been demonstrated to be a liver cancer suppressor [24, 47]. We showed that levels of CBX7 protein were significantly increased in GKO tumours compared to WT tumours (Fig. 5D, E). To further examine whether CBX7 upregulation could actually be induced by miR-181ab1 KO, we treated iKO mice with DEN to form primary liver tumours from which we then established primary liver tumour cell lines. In these iKO liver tumour cells, miR-181ab1 could be knocked out with 4-hydroxy-tamoxifen (4-OH-TAM) (supplementary data, Fig. S2B). We found levels of CBX7 protein were increased in 4-OH-TAM treated iKO primary liver tumour cells compared with their parental cells or 4-OH-TAM-treated Cre negative primary liver tumour cells (Fig. 5F, G), confirming miR-181ab1 KO up-regulated CBX7 expression.

CBX7 is a component of the polycomb repressive complex 1 (PRC1). It usually functions by inhibiting gene expression [23, 48, 49]. We presumed that CBX7 target genes, at least some CBX7 target genes, would be down-regulated in miR-181 KO tumours compared to WT tumours if CBX7 was functional. We collected CBX7 targets based on whole-genome ChIP-sequencing data (Supplementary data, Table S3) and compared these CBX7 target genes with 532 down-regulated genes in KO tumours compared with WT tumours [49, 50]. We found that 3.8% of top down-regulated DEGs in GKO tumours were CBX7 targets, including Wnt10a, CCNE1, FGFR2, and DUSP4 (Figs. 3B and 5H). Due to the limited availability of suitable antibodies, only some of them could be validated with western blotting on liver tumour tissues, confirming that Wnt10a and FGFR2 were down-regulated in GKO compared with WT liver tissues (Fig. 5I, J). Thus, CBX7 was identified as a strong candidate for mediating the effects of miR-181ab1 on liver tumour progression.

### Overexpression of CBX7 inhibited liver tumour progression

**Fig. 6:**
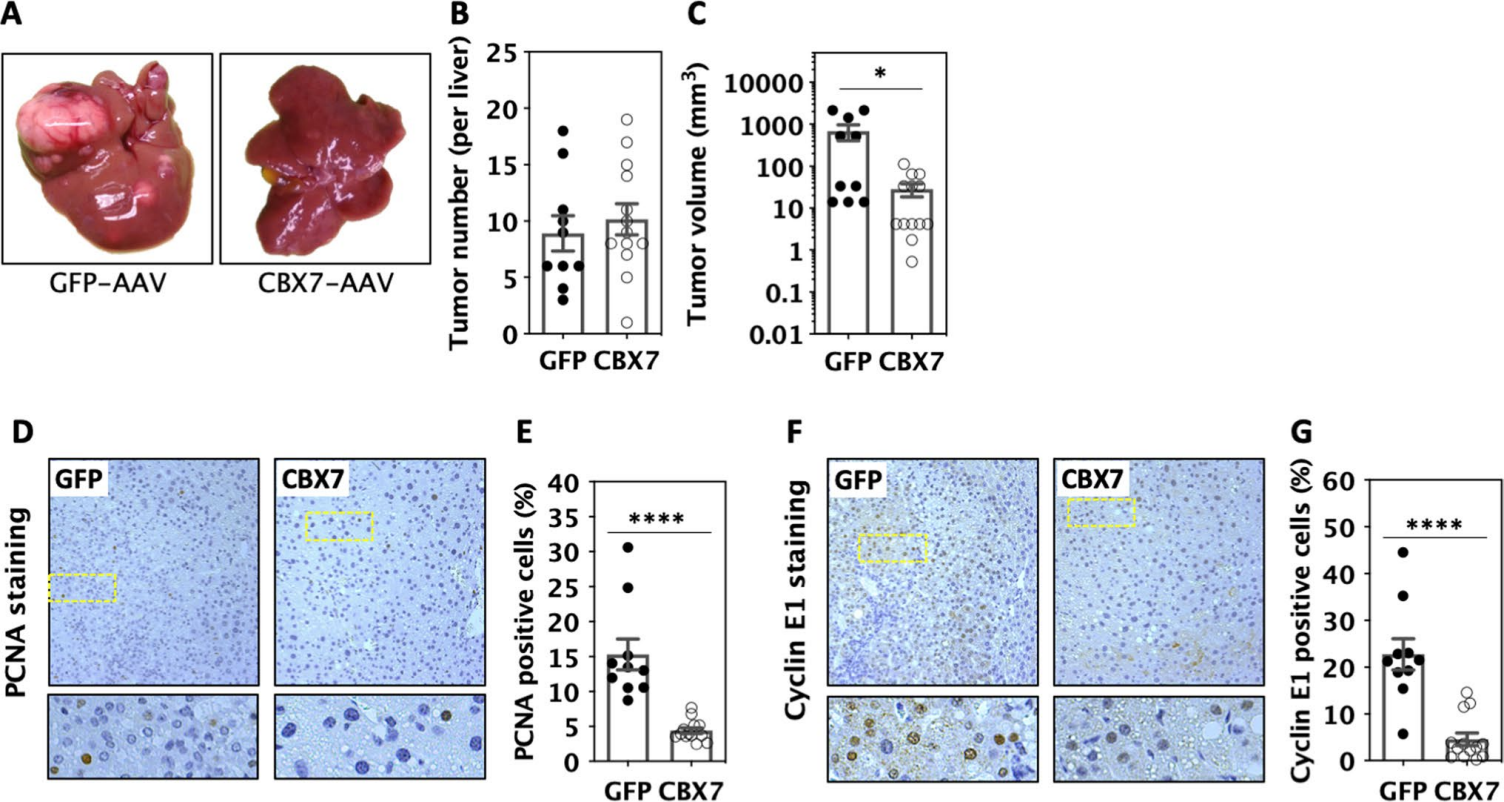
CBX7 expression inhibited liver tumour progression in vivo. DEN-treated mice received rAAVs at week 20 post-DEN and were harvested at week 34 post-DEN injection. **A** Macrographs of livers with tumours. GFP-AAV: controls, CBX7-AAV: treatments. **B** Liver tumour number in mice treated with CBX7-AAV and GFP-AAV. **C** Maximum size of liver tumours in CBX7-AAV and GFP-AAV-treated mice. **D** Micrographs and **E** quantifications of PCNA immuno-histochemical (IHC) staining. **F** Micrographs and **G** quantifications of Cyclin E1 IHC staining. Data were analysed by unpaired t test after data transformation if needed, ****p < 0.0001

The role of CBX7 expression in tumours is not clear. It has been linked to inhibition of primary tumour progression in vivo once tumours were already established, but also plays an oncogenic role in some cancers [51, 52]. Thus, we examined whether increased stable expression of CBX7 by rAAV with piggyBac transposase-mediated somatic integration in established primary WT tumour cells could inhibit tumour progression in vivo. Stable expression of CBX7 was evidenced in the majority of tumour cells at the end of experiments (Supplemental data, Fig. S5). CBX7-AAV treatment dramatically reduced the size of primary liver tumours by 96% compared with controls, i.e. GFP-AAV, in mice (Fig. 6A, C) without an alteration in the number of tumours (Fig. 6B). Furthermore, increased CBX7 greatly reduced tumour proliferation as measured by PCNA (Fig. 6D, E), and one CBX7 target, cyclin E1, which is essential for liver cancer progression [53, 54], was significantly reduced upon increased CBX7 expression (Fig. 6F, G).

In summary, WT tumours showed lower levels of CBX7 expression than miR-181ab1 KO tumours (Fig. 5D, E) and restored- or over-expressed CBX7 repressed liver tumour progression in WT mice (Fig. 6A–C). Thus, down-regulation of CBX7 was essential for the progression of liver tumours.

### CBX7 deletion restores progression of miR-181 KO primary liver tumours

**Fig. 7:**
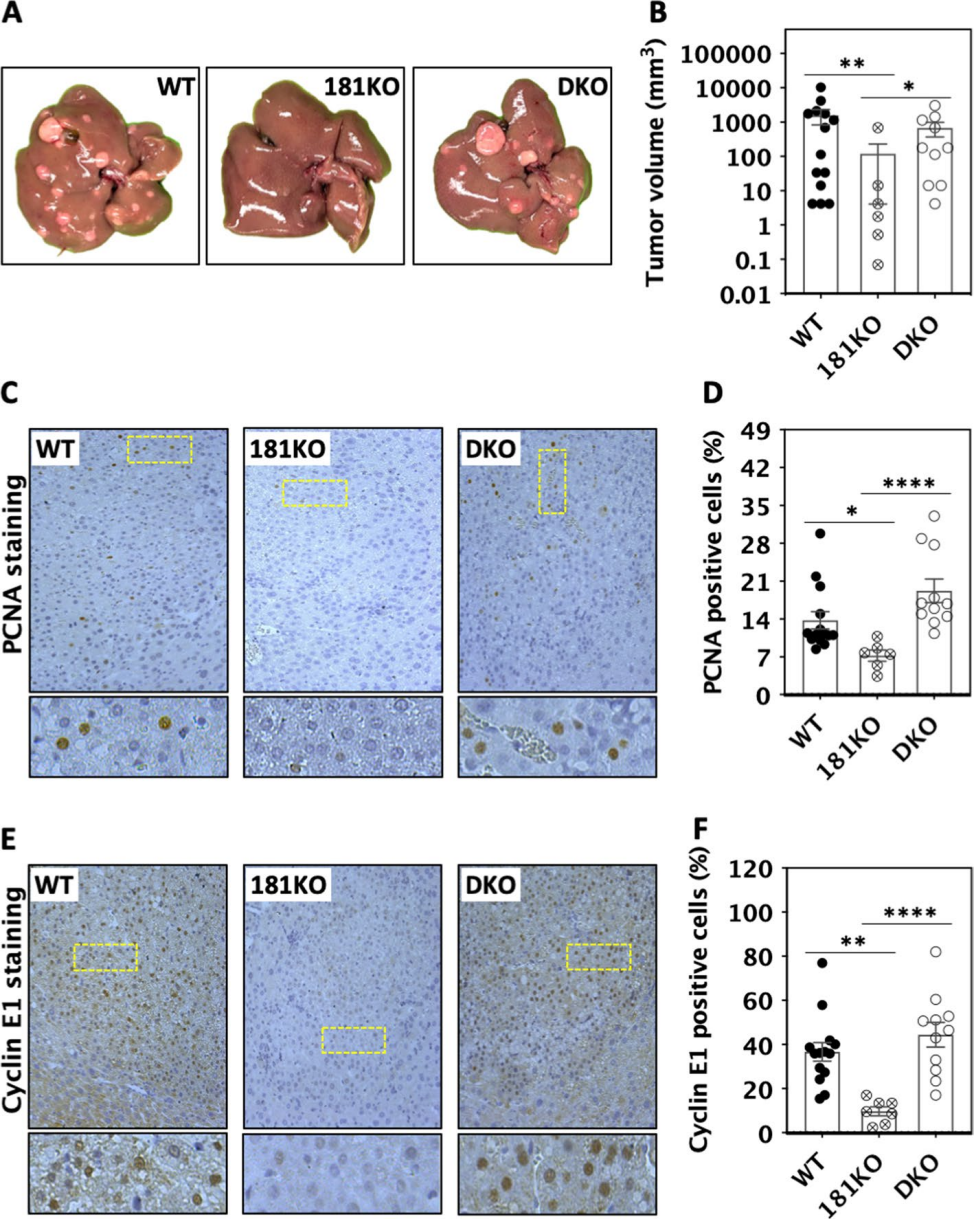
CBX7 deficiency restored the progression of miR-181 KO liver tumours. Non-tumourous liver and tumour samples were harvested at week 34 post-DEN injection. **A** Macrographs of livers with tumours. 181KO: hepatocyte-specific miR-181 KO; DKO: hepatocyte-specific miR-181 and CBX7 KO. **B** Maximum size of liver tumours. **C** Micrographs and **D** quantifications of PCNA immuno-histochemical (IHC) staining. **E** Micrographs and **F** quantifications of Cyclin E1 IHC staining. Data were analysed by Kruskal–Wallis, *p < 0.05, **p < 0.01, ****p < 0.0001

To address whether the miR-181-CBX7 pathway was critical for liver tumour progression, liver tumours were induced with DEN in liver-specific miR-181ab1 and CBX7 double knockout (DKO) mice and their progression was examined. As expected the size of liver tumours in LKO was reduced compared with WT mice at 34-week post-DEN administration (Fig. 7A, B). The size of liver tumours in the DKO mice was restored to a similar level to that seen in WT mice (Fig. 7A, B). Expressions of both cyclin E1 and PCNA in DKO tumours were also increased to a similar level as that of WT tumours (Fig. 7C–F). Thus, these results confirmed that up-regulation of CBX7 was essential for liver tumour inhibition in miR-181ab1 KO mice.

### The miR-181-CBX7 axis in human HCC

**Fig. 8:**
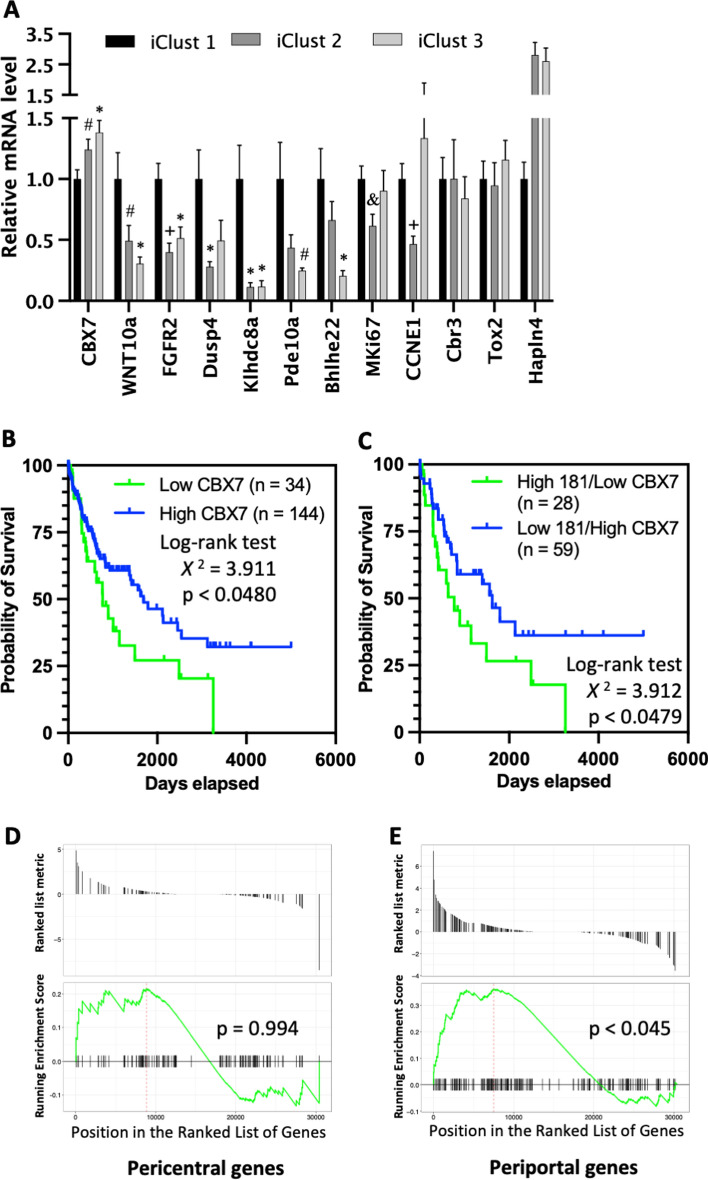
Relevance of miR-181/CBX7 in human HCC. **A** Expression levels of CBX7 targets in three iCluster subclasses of TCGA liver cancer. Brown-Forsythe and Welch ANOVA tests, compared to iClust1 of the same gene, #*p* < 0.05, & *p* < 0.01, *p < 0.005, + *p* < 0.0005. **B** Correlation of tumour CBX7 expression levels with survival of human HCC patients. **C** Correlation of tumour miR-181 expression with survival of human HCC patients. Results of computing overlaps between miR-181 loss-up-regulated genes and the **D** pericentral or **E** periportal hepatocyte gene set

It has been previously shown that miR-181a expression in human HCC is increased in iClust1 compared with iClust2 or iClust3 HCCs [4]. Thus, we analysed CBX7 expression in the TCGA liver cancer (TCGA-LIHC) data and found the iClust1 HCCs had significantly lower expression levels of CBX7 than either iClust2 or iClust3 HCCs (Fig. 8A). Expression levels of seven CBX7 targets, namely Wnt10a, Ccne1, Fgfr2, Bhlhe22, Klhdc8a, Pde10a, and Dusp4, which had been significantly inhibited in miR-181ab1 KO compared to WT tumours in mice (Fig. 5H), were also significantly lower in either iClust2 or iClust3 than iClust1 HCCs (Fig. 8A). Thus, the expression of miR-181, CBX7, and CBX7 targets follows patterns of expression within human HCC subtypes consistent with regulation of a miR-181/CBX7 axis. In addition, TCGA-LIHC survival data showed that HCC patients with the highest level of CBX7 mRNA (either alone or in combination with miR-181 levels) had significant longer survival than other patients (Fig. 8B, C).

Apart for the iCluster sub-classification of human HCC, other broader subtypes have been also analysed. These include the proliferative and non-proliferative subclasses [1, 55]. Non-proliferative HCCs that have a better outcome have been further subclassed into periportal-type HCC (ppHCC) and perivenous-type HCC (pcHCC) [55]. The ppHCC represents 29% of all HCCs and expresses wild-type b-catenin, has low TP53 mutation rate, and has the lowest potential for early recurrence after curative resection. We postulated that our low-proliferative miR-181 KO liver tumours may resemble the ppHCC subtype, as miR-181ab1 KO tumours showed increased expression of some genes, such as CBX7 and FOXP2, which were mainly expressed in periportal hepatocytes (periportal genes) (Supplemental data, Fig. S6A-B; Fig. 5D, E). To this end, we compared genes up-regulated by miR-181 KO with a set of periportal genes and confirmed miR-181 KO tumours had enriched expression of periportal genes (Fig. 8E), but not genes that are mainly expressed in pericentral hepatocytes (pericentral genes) (Fig. 8D).

## Discussion

This study provides many valuable and novel findings. First, miR-181ab1 is a key driver of liver tumour growth. Second, there is a clear link between miR-181 and CBX7, which is critical for mediating the effects of miR-181 on liver tumour progression. Finally, miR-181ab1-deficient liver tumours have low proliferation rates.

Consistently, miR-181 has been identified as a potential promoter of HCC development in several studies [16, 18, 19, 22]. For example, Wang et al. reported hepatic miR-181b promotes choline-deficient and L-amino acid-defined (CDAA)-induced liver cancer development [19]. Ji et al. showed miR-181 was a critical player in EpCAM-positive hepatic cancer stem cells [18]. Our results, for the first time, clearly show that hepatic miR-181 is involved in tumour growth and progression using several tissue-specific miR-181ab1 knockout mice. Hepatic miR-181 mediation of HCC tumour growth was also supported by in vitro experiments and other literature using cell lines in other models [16, 22, 40]. On the other hand, only GKO had suppressive effects on the initiation of tumours, suggesting miR-181 KO in an unknown cell population or multiple cell populations may be required to restrict tumour initiation.

Identification of miR-181 targets responsible for the effects of miR-181 on liver tumour progression is vital not just for understanding how miR-181 KO reduces liver tumour progression but also for discovering new therapeutic targets. In combination with other studies, the current study demonstrated that CBX7 as a conserved tumour suppressor mediated the effects of miR-181 on liver tumour progression. First, CBX7 has been identified by several independent studies to be a miR-181 target [23, 24]. Second, the deletion of miR-181 leads to an increase in CBX7 expression in vitro and in vivo. Third, CBX7 overexpression inhibited liver tumour progression, supporting findings by Forzati et al. who demonstrated CBX7 loss caused spontaneous liver cancer formation in mice [56]. Fourth, CBX7 knockout restored miR-181 loss-induced liver tumour inhibition. Progression of DEN-induced liver tumours was similar between wild type and CBX7 single KO mice, largely indicating that CBX7 deficiency did not override any other type of suppressive effect (Supplemental data, Fig. S7). Finally, miR-181 and CBX7 were inversely expressed in human HCC [22, 57]. Thus, these compelling pieces of evidence strongly support CBX7 playing a major role in mediating the effect of miR-181 on liver tumour progression.

CBX7 is a component of the Polycomb repressive complex 1, which maintains the transcriptionally repressive state of many genes via chromatin remodelling and modification of histones [47, 50, 58]. CBX7 interacts with diverse regulatory proteins and thus alters the expression of its direct downstream targets [58, 59]. CBX7-mediated tumour suppression is in part through inhibition of of cyclin E1 [56, 60]. This notion was strongly supported in this study, but cyclin E1 expression in iClust3 HCCs (with low miR-181 and high CBX7 expressions) is not lower than that in iClust1 HCCs. Unfortunately, we could not explore this relationship in another human HCC database due to the lack of expression profiles of microRNAs and CBX7. In addition, we showed that several other CBX7 targets were down-regulated at the transcriptional level in both miR-181 KO mouse liver tumours and iClust3 human HCCs. Low levels of FGFR2 and WNT10a protein were confirmed. However, more studies of the miR-181/CBX7 axis are needed, especially on human HCC samples, to thoroughly examine the roles of CBX7 targets and partners (such as cyclin E1, HDAC2, and TWIST) in HCC progression and their underlying mechanisms [56, 58, 59, 61, 62].

Primary liver tumours still formed in GKO mice. These miR-181ab1 KO tumours had lower proliferative rate and primary liver tumour cells derived from miR-181ab1-deficient tumours still can grow in vitro, though much slower than WT tumour cells. Indeed, genes associated with human periportal-type low-proliferative HCCs were enriched in miR-181 knockout tumours. It raises the possibility that further investigations of miR-181 knockout tumours could serve as a model to study therapies in the low-proliferative HCC subtype, which is currently under-represented in HCC cell lines [63]. Thus, a miR-181ab1-deficient liver tumour mouse model may also have significant implications for the study of low-proliferative human HCC.

This study has several limitations. First, the miR-181/CBX7 axis clearly played a vital role in DEN-induced liver cancer animal models (the DEN model), but this may not always be the case in other liver cancer animal models. For example, TIMP3 was reported to mediate promoting effects of miR-181b on tumour progression in CDAA-induced liver cancer in which TIMP3 was down-regulated [19]. In contrast, TIMP3 expression was increased in human HCC compared with normal tissue [45]. TIMP3 expression was also consistently increased in DEN-induced liver tumours compared with non-tumour livers. Expression levels of TIMP3 were similar between DEN-induced miR-181 KO and WT tumours in mice (data not shown). Thus, the effect of miR-181 in different tumours with different genetic changes may be mediated by diverse genes [22, 64]. The DEN model lacks some vital features in human liver cancer development, such as chronic liver inflammation and liver fibrosis. Thus, additional animal models, especially models with chronic liver inflammation and liver fibrosis, may be needed to further explore the miR-181/CBX7 axis in hepatocarcinogenesis [65–67]. Second, the miR-181/CBX7 axis might play a role in some human HCCs only, as only one-third of human HCCs show high miR-181 and low CBX7 expressions [4]. Thus, it is vital to analyse more human samples or databases other than TCGA to fully understand the connection between miR-181 and CBX7 in different subtypes of human HCC.

Furthermore, one of the issues not addressed in this study was the potential mechanisms responsible for increased miR-181 expression in HCC. First, we and others have shown that TGF-beta increased miR-181 expression and EMT in hepatocytes and promoted tumour progression [16, 40, 68]. The current study showed that miR-181ab1 loss altered the expression levels of TGF-beta signature genes, suggesting miR-181 might also regulate the TGF-beta pathway. This notion has been confirmed in squamous cell carcinoma [69]. However, further investigations are needed to examine detailed aspects of molecular regulations between miR-181, the TGF-beta pathway, and EMT. Second, others have shown that the WNT/beta-catenin signalling pathway transcriptionally activated miR-181 expression in human HCC [70]. In contrast, we found liver tumours had similar expression levels of active beta-catenin protein in DEN-treated GKO and WT mice (data not shown), indicating miR-181 does not regulate the activity of beta-catenin.

## Conclusion

Liver-specific deletion of miR-181ab1 significantly inhibited the progression, but not initiation of DEN-induced liver tumours in mice. The effects of miR-181ab1 deficiency on tumour progression were mediated by CBX7. The miR-181-CBX7 axis plays a vital role in DEN-induced mouse liver cancers and probably in iClust1 human HCCs, offering new potential therapeutic targets for some subtypes of HCCs. MiR-181 deficient liver tumours may represent the non-proliferative peri-portal HCC subtype. Therefore, miR-181ab1 knockout mice may be of use as a new animal model to study therapeutic intervention in this subclass of human HCC.

### Supplementary Information

Below is the link to the electronic supplementary material.

Supplementary file1 (DOCX 95540 KB)

## Data Availability

The RNA-seq datasets generated during the current study are available in the GEO repository, https://www.ncbi.nlm.nih.gov/geo/query/acc.cgi?acc=GSE196008.
