# Supplementary material for: Liver-specific deletion of miR-181ab1 reduces liver tumour progression via upregulation of CBX7
Source: Cell Mol Life Sci. 2022 Jul 22;79(8):443. doi: 10.1007/s00018-022-04452-6 (PMC9307539; doi:10.1007/s00018-022-04452-6)
Supplement: Supplementary file 1 — Supplementary file1 (DOCX 95540 KB) [file 18_2022_4452_MOESM1_ESM.docx]

**Supplemental Figure S1**


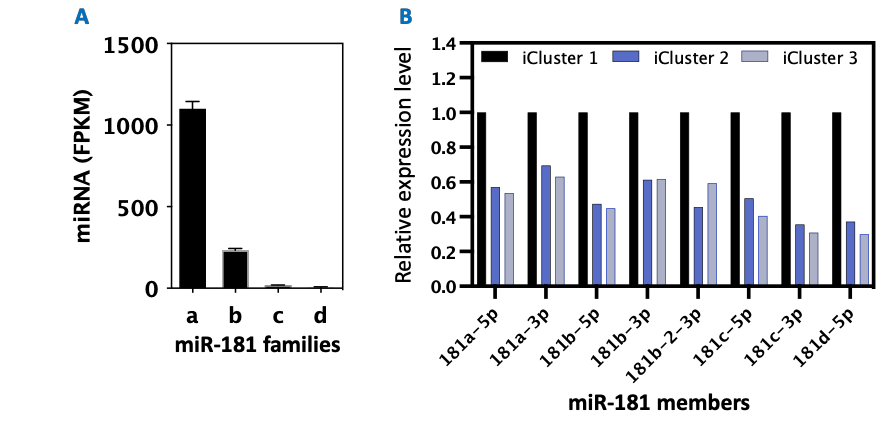


**Fig. S1. MiR-181 in human HCC.** TCGA liver cancer data were either downloaded from OncoLnc website or a paper published in Cell (doi: 10.1016/j.cell.2017.05.046). **(A)** Levels of miR-181 families in human HCC. **(B)** Levels of miR-181 members in each iCluster.

**Supplemental data**

**Supplemental experimental procedures**

**Materials**

Diethylnitrosamine (cat # N0258), collagenase IV (cat # C5138), hydrocortisone (cat # H2270), insulin (cat # I9278), and 4-Hydroxytamoxifen (4-OHT, cat # H7904) were purchased from Sigma–Aldrich (Sydney, Australia). Antibody against CBX7 was ordered from Millipore/Mark (cat # 07-981). Antibodies against CBX7 (cat # ab21873), FOXP2 (cat # ab16046), DUSP4 (cat # ab216576), WNT10a (cat # ab106522), beta-actin (cat # 197277), and GAPDH (cat # ab9482) were bought from Abcam (Cambridge, CB4 0FL, UK). Antibodies against PCNA (cat # 13110s), CDH1 (cat # 3195s), cyclin E1 (cat # 20808), cyclin D1 (cat # 2978), Snail (cat # 3879), Slug (cat # 9585), CDH2 (cat # 4061), and FGFR2 (cat # 23328) were obtained from Cell Signaling Technology (Danvers, MA, USA).

**Supplemental experimental procedures**

**Mouse genotyping**

PCR genotyping protocol was used to genotype Cre-positive and flox mice. Primers were provided by the donating investigator of strains (Supplemental data, Table S1). Genomic DNA was prepared from ear tissues as reported previously ^[^[^1^](#_ENREF_1)^]^. Examples of genotyping results were shown in Figure S2.

**Table S1 Primers used in the genotyping**


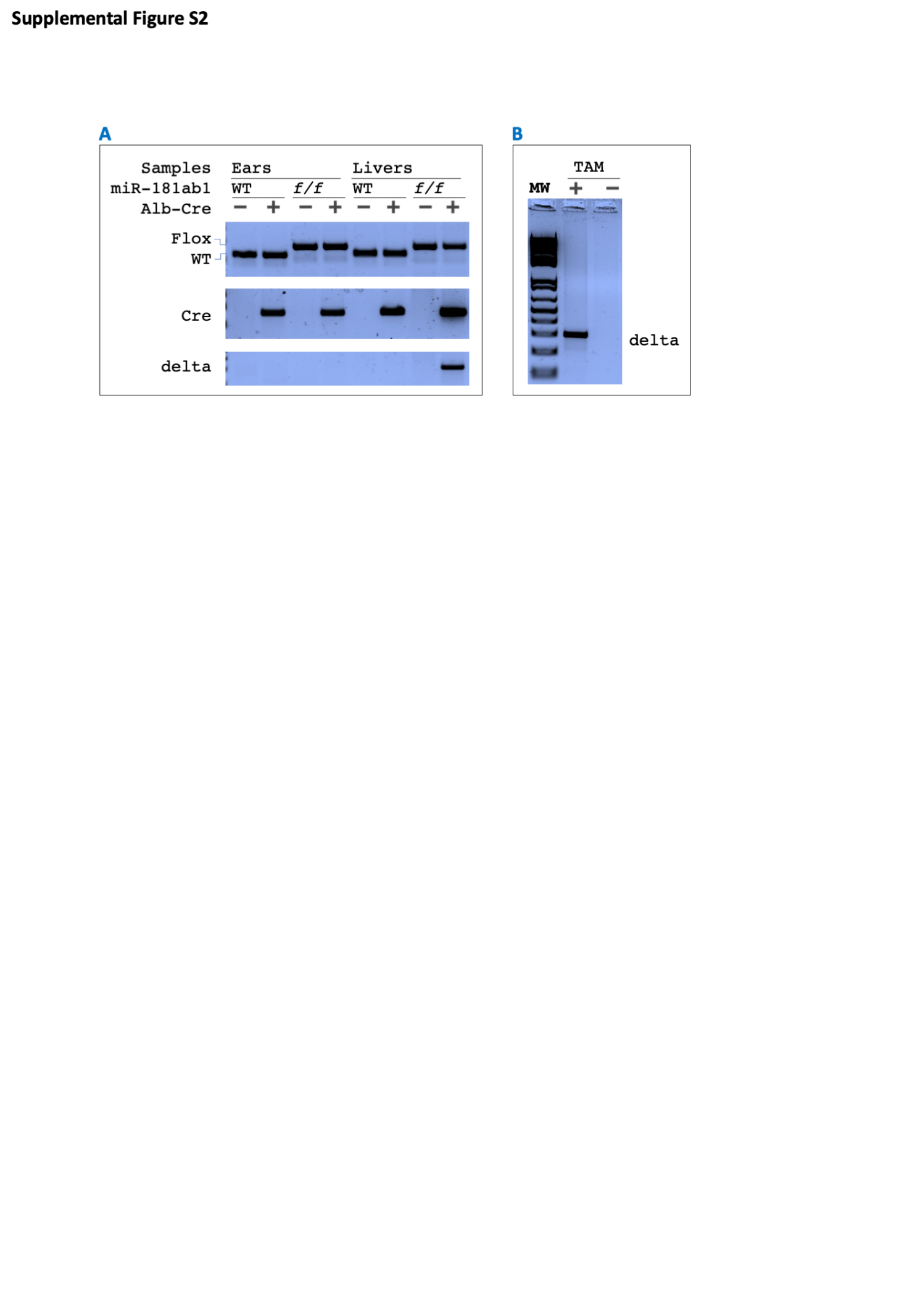
**Supplemental Figure S2**

**Fig. S2. Genotyping examples of miR-181ab1 knockout mice.** Ear punches, liver tissues, or liver tumour cells were collected. Quick DNA purification protocol from the Jackson Laboratory was used to extract DNA. Three pairs of primers were used to detect flox, Cre and deletion (delta) samples. **(A)** In miR-181ab1^f/f^:AlbCre mice: PCR products of miR-181ab1 deletion were detected in livers only, not ears, of floxed and Cre positive mice. **(B)** In miR-181ab1^f/f^:R26Cre^ER^ mice or isolated liver tumour cells: PCR products of miR-181ab1 deletion were detected after the tamoxifen (TAM) treatment. MW: molecular weight.

**DEN-induced liver cancer mouse model**

The DEN-induced primary liver cancer mouse model has been previously reported ^[^[^2^](#_ENREF_2)^]^. In brief, DEN (25 mg/kg) was injected once intraperitoneally (i.p.) into male pups at postnatal day 12~14. Wild type and gene knockout littermates were housed in the same cage (2~6 mice per cage), and were euthanized by CO_2_ inhalation at 19- and 34-week post-DEN injection. Visible tumours (≥0.5mm) on the surface of each liver were counted and their size was measured with a calliper without knowing the genotype, which was examined after DEN injection or even after mice were euthanized. The tumour volume was calculated with the formula for the volume of a sphere. Tumour tissues, their surrounding non-tumour tissues and age-matched healthy liver tissues were collected and either fixed in 10% neutral buffered formalin or snap frozen for further analysis.

**Histopathological analysis**

Paraffin tissue sections (4μm) of livers were cut and stained with haematoxylin and eosin (H&E). Early stage tumours were identified and quantified on H&E sections as described previously ^[^[^2^](#_ENREF_2)^]^. Briefly, the number of foci per liver section and the area of each liver section were measured and results expressed as the number of foci per mm^2^.

The immunohistochemistry used to detect protein expression of genes and TUNEL (Roche) staining used to detect apoptosis were described previously ^[^[^2^](#_ENREF_2)^]^. The percentage of TUNEL-, PCNA- and cyclin D1-positive cells were quantified with the help of FIJI software. A Semi-quantitative immuno-histochemical assay was applied to expression of CBX7 and other factors, i.e. the intensity of staining was graded as negative (0), weak (1), moderate (2) and strong (3).

**Western blotting**

The level of protein expression was determined with Western blotting as described previously ^[^[^3^](#_ENREF_3)^]^.

**Reference:**

[1] Truett GE, Heeger P, Mynatt RL, Truett AA, Walker JA, Warman ML. Preparation of PCR-quality mouse genomic DNA with hot sodium hydroxide and tris (HotSHOT). Biotechniques 2000;29:52, 54.

[2] Chen J, Qi Y, Zhao Y, Kaczorowski D, Couttas TA, Coleman PR, et al. Deletion of sphingosine kinase 1 inhibits liver tumorigenesis in diethylnitrosamine-treated mice. Oncotarget 2018;9:15635-15649.

[3] Qi Y, Wang W, Chen J, Dai L, Kaczorowski D, Gao X, et al. Sphingosine Kinase 1 Protects Hepatocytes from Lipotoxicity via Down-regulation of IRE1alpha Protein Expression. J Biol Chem 2015;290:23282-23290.

**Table S2 DEGS overlap with liver cancer gene sets in MSigDB**

**Supplemental data, Table S2**


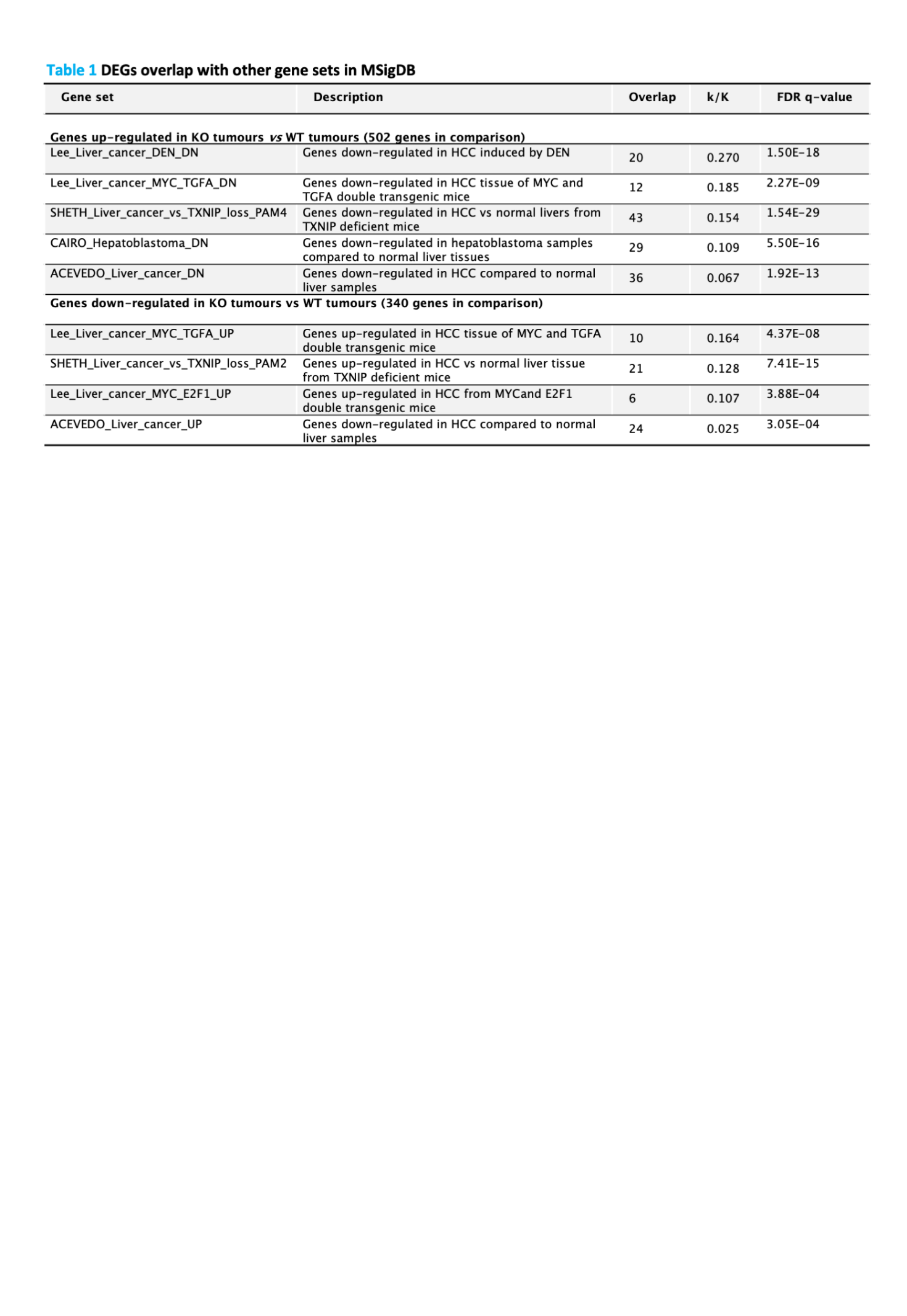


**Supplemental Figure S3**


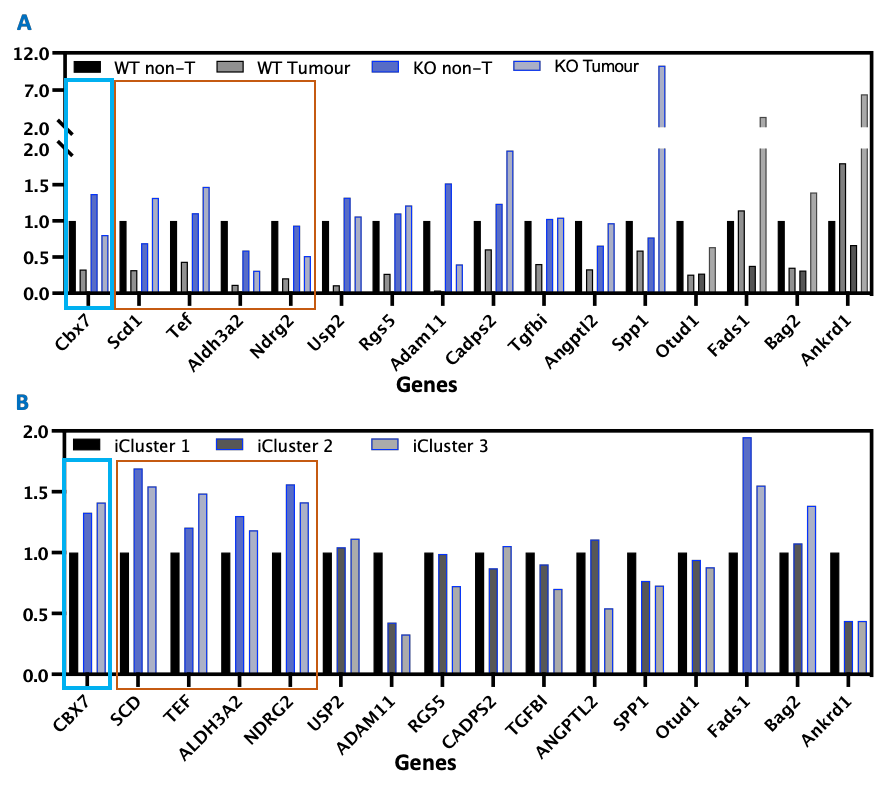


**Fig. S3. Differentially expressed miR-181 target genes.** There are sixteen top up-regulated miR-181 target genes in miR-181ab1 knockout (KO) tumours compared to wild-type (WT) tumours. **(A)** Relative mRNA levels of top up-regulated genes in non-tumour (non-T) and tumour tissues harvested from mice at week 34 post-DEN injection. 3 mice each group. **(B)** Relative mRNA levels of genes in iCluster 1, 2 and 3 human HCC.

**Supplemental Figure S4**


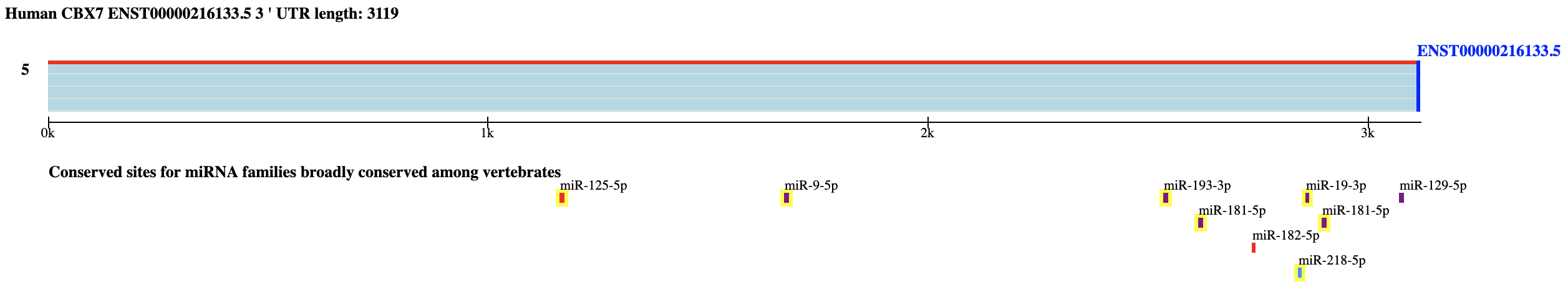

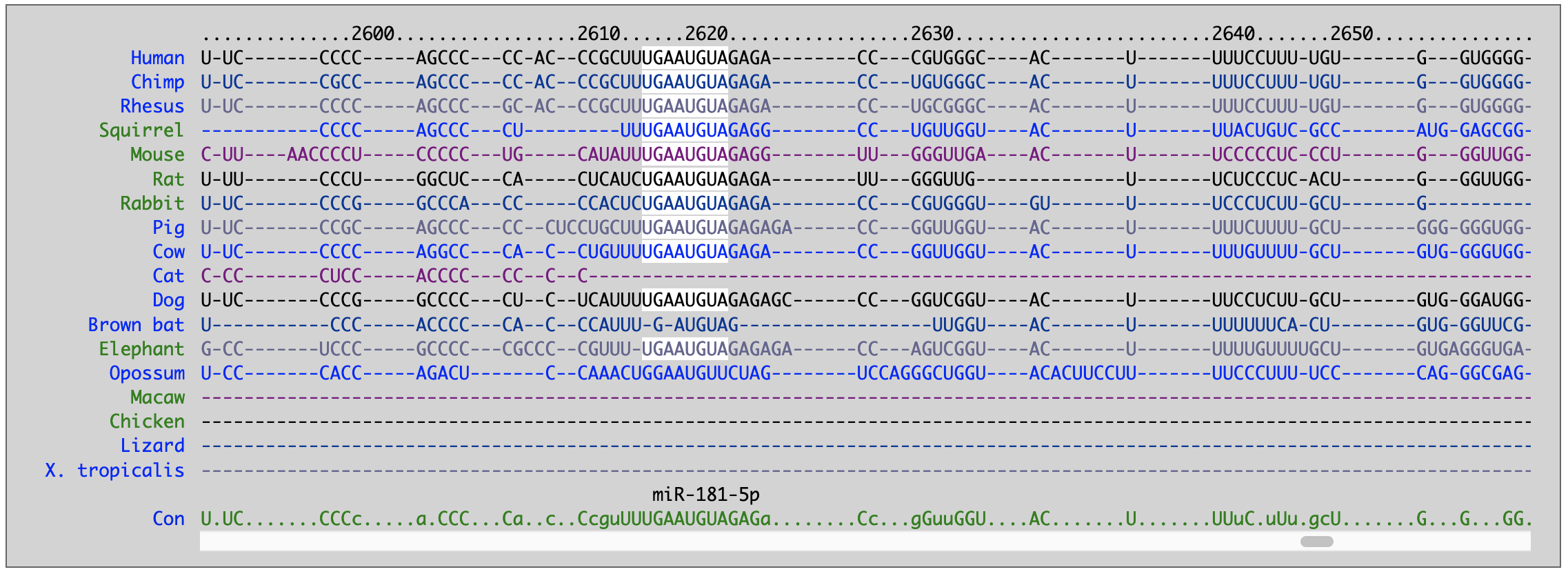

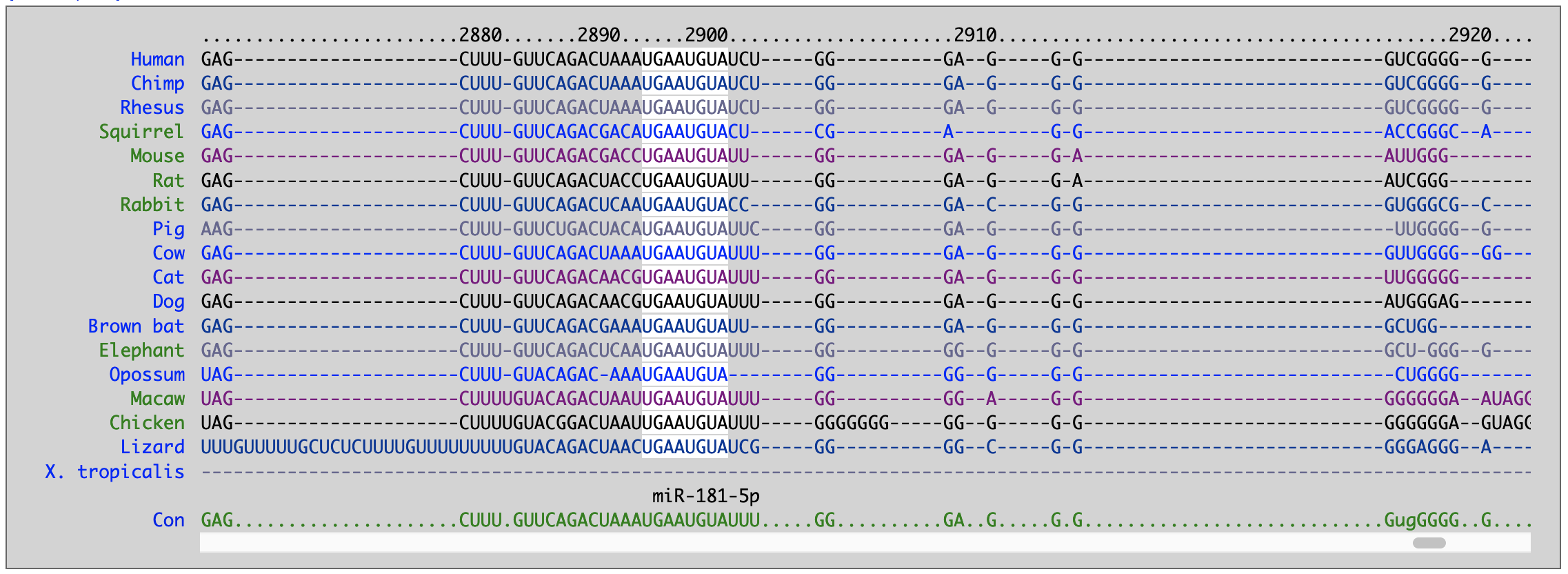


**Fig. S4. miR-181 target sites in CBX7.** There are two miR-181 target sites in the 3’ UTR of CBX7 according to TargetScan. There are around 300bp between two target sites. Both target sites are conserved among mammals. Agarwal V, et al. eLife 2015;4:e05005 doi: : 10.7554/eLife.05005. McGeary SE, et al. Science. 2019 Dec 20;366(6472):eaav1741. doi: 10.1126/science.aav1741.

**Supplemental data, Table S3**

| **common CBX7 target genes** | | | | | | | | | |
| --- | --- | --- | --- | --- | --- | --- | --- | --- | --- |
| 1110032F04Rik |  | Bcan |  | Dbndd2 |  | Fgf18 |  | Gucy2e |  |
| 1300014I06Rik |  | Bcor |  | Dbx1 |  | Fgf3 |  | H2-Ab1 |  |
| 1700003E16Rik |  | Bend7 |  | Ddn |  | Fgf8 |  | H2afy2 |  |
| 1700109F18Rik |  | Bhlhe22 |  | Des |  | Fgf9 |  | H60b |  |
| 2010300C02Rik |  | Bhlhe41 |  | Dhh |  | Fgfr2 |  | Hand2 |  |
| 2510009E07Rik |  | Bmi1 |  | Dlgap1 |  | Fgfr3 |  | Hapln4 |  |
| 2810030E01Rik |  | Bmp1 |  | Dlgap2 |  | Fhdc1 |  | Hecw2 |  |
| 2810459M11Rik |  | Bmp8a |  | Dlgap3 |  | Fibcd1 |  | Heg1 |  |
| 4732444A12Rik |  | Bmp8b |  | Dll1 |  | Fjx1 |  | Hes2 |  |
| 4921522P10Rik |  | Bmper |  | Dll4 |  | Flrt2 |  | Hey2 |  |
| 4931408A02Rik |  | Boc |  | Dlx1 |  | Flt1 |  | Hhipl1 |  |
| 4932442L08Rik |  | C130021I20Rik |  | Dlx2 |  | Foxa1 |  | Hic1 |  |
| 5033430I15Rik |  | C130071C03Rik |  | Dlx4 |  | Foxb2 |  | Hivep3 |  |
| 5430405G05Rik |  | C1qtnf4 |  | Dmbx1 |  | Foxc1 |  | Hmga2 |  |
| 8430427H17Rik |  | C530008M17Rik |  | Dmrta1 |  | Foxc2 |  | Hmx2 |  |
| 9130019P16Rik |  | Cacna1c |  | Dmrta2 |  | Foxd1 |  | Hnf1b |  |
| 9830001H06Rik |  | Cacng4 |  | Dnahc8 |  | Foxd2 |  | Hoxa1 |  |
| A930004D18Rik |  | Camk2b |  | Dok6 |  | Foxd3 |  | Hoxa10 |  |
| AB041803 |  | Cap2 |  | Dpp6 |  | Foxd4 |  | Hoxa11 |  |
| Abhd6 |  | Casz1 |  | Drd1a |  | Foxe1 |  | Hoxa13 |  |
| Abtb2 |  | Cbfa2t3 |  | Drd4 |  | Foxf2 |  | Hoxa7 |  |
| Accn2 |  | Cbr3 |  | Dscam |  | Foxl2 |  | Hoxb7 |  |
| Acot6 |  | Cbx8 |  | Duox2 |  | Foxo6 |  | Hoxc11 |  |
| Adamts17 |  | ccne1 |  | Duoxa1 |  | Frmd4a |  | Hoxc12 |  |
| Adm |  | Ccr10 |  | Duoxa2 |  | Fryl |  | Hoxc5 |  |
| Adora1 |  | Cdh13 |  | Dusp4 |  | Frzb |  | Hoxc9 |  |
| Adora2a |  | Cdh23 |  | E130114P18Rik |  | Fstl4 |  | Hoxd11 |  |
| Adra1b |  | Cdh4 |  | E2f6 |  | Fzd1 |  | Hoxd12 |  |
| Adra2a |  | Cdkn2a |  | Ebf3 |  | Fzd2 |  | Hoxd13 |  |
| Aff3 |  | Chat |  | Ebf4 |  | Fzd9 |  | Hoxd3 |  |
| AI464131 |  | Chst1 |  | Ece2 |  | Gab1 |  | Hoxd9 |  |
| AI854703 |  | Chst2 |  | Ecel1 |  | Gab2 |  | Hspa1a |  |
| Ak5 |  | Cilp2 |  | Efhd1 |  | Gabbr2 |  | Hspa1b |  |
| Alk |  | Cir1 |  | Efnb2 |  | Gal3st1 |  | Hspa1l |  |
| Alx3 |  | Cldn23 |  | Egr3 |  | Galnt6 |  | Htra1 |  |
| Alx4 |  | Clec2l |  | Eif4g3 |  | Gata2 |  | Htra4 |  |
| Ank1 |  | Cnih3 |  | Elfn1 |  | Gata4 |  | Ica1l |  |
| Ankrd33b |  | Cnnm1 |  | Emid2 |  | Gbx1 |  | Id3 |  |
| Ankrd43 |  | Cntnap1 |  | Eml6 |  | Gdf6 |  | Id4 |  |
| Ano1 |  | Coch |  | En1 |  | Gdf7 |  | Igdcc3 |  |
| Antxr1 |  | Col13a1 |  | Enpp1 |  | Gjc2 |  | Igdcc4 |  |
| Apoo |  | Col27a1 |  | Eomes |  | Gkap1 |  | Igf1r |  |
| Aqp2 |  | Col4a1 |  | Epas1 |  | Gm1568 |  | Igf2bp2 |  |
| Aqp5 |  | Col4a2 |  | Epb4.1 |  | Gm1614 |  | Ihh |  |
| Arl4c |  | Comp |  | Epb4.1l4b |  | Gm8909 |  | Ikzf3 |  |
| Arpp21 |  | Cpm |  | Epha7 |  | Gm996 |  | Il11ra1 |  |
| Artn |  | Cpne5 |  | Ephb1 |  | Gnal |  | Ildr2 |  |
| Atoh8 |  | Crim1 |  | Erg |  | Gp1bb |  | Insm1 |  |
| Atp2b2 |  | Crlf1 |  | Espn |  | Gpr150 |  | Irx4 |  |
| Atp8b1 |  | Crtc3 |  | Esr2 |  | Gpr176 |  | Isl1 |  |
| Atxn1 |  | Cxcl14 |  | Evx1 |  | Gpr25 |  | Isl2 |  |
| B3galnt2 |  | Cybrd1 |  | Evx2 |  | Gpr88 |  | Islr2 |  |
| B3gat2 |  | Cygb |  | Fam174b |  | Grb10 |  | Ism1 |  |
| B3gnt6 |  | Cyp1b1 |  | Fam59b |  | Grin2c |  | Itpka |  |
| Bach2 |  | Cyp24a1 |  | Farp1 |  | Grin2d |  | Itpkb |  |
| Bai2 |  | Cys1 |  | Fat4 |  | Grip1 |  | Itpr2 |  |
| Bank1 |  | D230002A01Rik |  | Fbn1 |  | Grm4 |  | Kazald1 |  |
| Barhl2 |  | D930049A15Rik |  | Fbxl8 |  | Grrp1 |  | Kbtbd11 |  |
| Bbs9 |  | Dab2ip |  | Fev |  | Gsc2 |  | Kbtbd5 |  |
| BC061194 |  | Dact1 |  | Fgf15 |  | Gsk3b |  | Kcnh3 |  |
| Kcnj12 |  | Nav2 |  | Phldb2 |  | Sarm1 |  | Tbx2 |  |
| Kcnk13 |  | Nbea |  | Phox2a |  | Satb2 |  | Tbx4 |  |
| Kcnk15 |  | Nefh |  | Phox2b |  | Scarf2 |  | Tc2n |  |
| Kcnq5 |  | Nes |  | Pitx1 |  | Scn4b |  | Tcfap2a |  |
| Kcns1 |  | Neurod2 |  | Pitx2 |  | Scn8a |  | Tcfap2e |  |
| Kctd1 |  | Neurog2 |  | Pitx3 |  | Sdc1 |  | Tchh |  |
| Klhdc8a |  | Nfatc1 |  | Pknox2 |  | Sdk1 |  | Tdrd5 |  |
| Klhl31 |  | Nfia |  | Plekha2 |  | Sdk2 |  | Tesc |  |
| Klrg2 |  | Nfib |  | Plk5 |  | Sema3f |  | Tgfb2 |  |
| Lancl3 |  | Nin |  | Plxna4 |  | Sema5b |  | Thada |  |
| Larp1b |  | Nkd1 |  | Plxnd1 |  | Sema6b |  | Tlx2 |  |
| Lbx1 |  | Nkpd1 |  | Podxl |  | Setd7 |  | Tmeff2 |  |
| Lbx2 |  | Nkx2-1 |  | Pou4f1 |  | Shb |  | Tmem117 |  |
| Lef1 |  | Nkx2-3 |  | Ppp1r3g |  | Shisa3 |  | Tmem151a |  |
| Lhx1 |  | Nkx6-1 |  | Ppp2r2c |  | Shox2 |  | Tmem151b |  |
| Lhx2 |  | Nog |  | Prdm12 |  | Shroom1 |  | Tmem163 |  |
| Lhx4 |  | Notum |  | Prdm13 |  | Sim1 |  | Tmem30b |  |
| Lhx6 |  | Npas1 |  | Prdm16 |  | Sim2 |  | Tmem56 |  |
| Lhx8 |  | Npas2 |  | Prdm6 |  | Six1 |  | Tnfsf11 |  |
| Lhx9 |  | Npas3 |  | Prex2 |  | Six2 |  | Tnrc18 |  |
| Lingo1 |  | Nppc |  | Prhoxnb |  | Six3 |  | Tox2 |  |
| Lmx1b |  | Npr1 |  | Prickle1 |  | Slc10a4 |  | Tpm1 |  |
| Lonrf2 |  | Nr2e1 |  | Prkcdbp |  | Slc1a6 |  | Trim2 |  |
| Lor |  | Nr3c2 |  | Prkce |  | Slc22a23 |  | Trim62 |  |
| Lox |  | Nr4a2 |  | Prkch |  | Slc22a3 |  | Trp73 |  |
| Lrba |  | Nr4a3 |  | Prkg1 |  | Slc24a3 |  | Trpc3 |  |
| Lrp8 |  | Nr5a1 |  | Prok2 |  | Slc2a13 |  | Tshz2 |  |
| Ltbp2 |  | Nrxn1 |  | Prr7 |  | Slc32a1 |  | Tspan18 |  |
| Mab21l1 |  | Nt5e |  | Prss12 |  | Slc41a2 |  | Ttc28 |  |
| Mab21l2 |  | Ntn1 |  | Prtg |  | Slc6a17 |  | Uggt2 |  |
| Macrod1 |  | Ntn4 |  | Psd4 |  | Slc6a2 |  | Unc45b |  |
| Maf |  | Nxph4 |  | Ptf1a |  | Slc6a20a |  | Unc5b |  |
| Mafb |  | Obscn |  | Ptger3 |  | Slc9a2 |  | Uncx |  |
| Mal |  | Ocln |  | Pth1r |  | Slco5a1 |  | Vash2 |  |
| Maml3 |  | Olig2 |  | Ptpru |  | Slitrk5 |  | Vax2 |  |
| Man1a |  | Onecut3 |  | Rasd2 |  | Smad6 |  | Vsx2 |  |
| Map3k5 |  | Osbpl6 |  | Rasgrf1 |  | Smarca2 |  | Vwa5b2 |  |
| Mapk12 |  | Osmr |  | Rasgrf2 |  | Sncaip |  | Wars2 |  |
| Mapk15 |  | Osr2 |  | Rax |  | Sobp |  | Wipf3 |  |
| Mapt |  | Ostm1 |  | Rbm24 |  | Sox13 |  | Wnt10a |  |
| Mast4 |  | Otop1 |  | Rem1 |  | Sox21 |  | Wnt11 |  |
| Mctp1 |  | Otp |  | Ret |  | Sox6 |  | Wnt2b |  |
| Mdga1 |  | Otud7a |  | Rgl1 |  | Sox7 |  | Wnt3a |  |
| Mecom |  | Otx1 |  | Rgl3 |  | Sp5 |  | Wnt9a |  |
| Med12l |  | Otx2 |  | Rgs7bp |  | Sp6 |  | Wnt9b |  |
| Med20 |  | Ovol1 |  | Rgs9bp |  | Sp9 |  | Zar1 |  |
| Megf11 |  | Ovol2 |  | Ripk4 |  | Spata3 |  | Zbtb16 |  |
| Meis2 |  | Oxtr |  | Rnf150 |  | Speg |  | Zdhhc14 |  |
| Mex3b |  | P2ry1 |  | Rnf220 |  | Sphk1 |  | Zfp316 |  |
| Mfhas1 |  | Pacsin3 |  | Ror1 |  | Srrm3 |  | Zfp608 |  |
| Mfsd2a |  | Paqr9 |  | Rora |  | Ssbp2 |  | Zfp652 |  |
| Mfsd4 |  | Pax2 |  | Rprml |  | Stk39 |  | Zfp697 |  |
| Mgll |  | Pax6 |  | Rtn4r |  | Syn2 |  | Zhx2 |  |
| Mllt3 |  | Pcdh1 |  | Rtn4rl1 |  | Syne2 |  | Zic1 |  |
| Mmp15 |  | Pcdhga11 |  | Rtn4rl2 |  | Synm |  | Zic4 |  |
| Mn1 |  | Pde10a |  | Runx1 |  | Syt12 |  | Zic5 |  |
| Mnx1 |  | Pde4b |  | Runx3 |  | Syt2 |  | Znrf4 |  |
| Mocos |  | Pdx1 |  | S1pr5 |  | Tal1 |  | Zswim6 |  |
| Mrc2 |  | Peli2 |  | Sall3 |  | Tbc1d30 |  |  |  |
| Myrip |  | Penk |  | Samd4 |  | Tbr1 |  |  |  |
| Nat8l |  | Pfdn4 |  | Samd5 |  | Tbx15 |  |  |  |

**Supplemental Figure S5**

**Fig. S5. Long-term expression of genes delivered with recombinant AAV vectors in mouse livers.** Control GFP and CBX7 were delivered into mouse livers with the hybrid rAAV‐piggyBac gene therapy system, but the transposase-GFP was delivered with the conventional AAV system. Livers were harvested after 14 weeks of AAV administration (C~F). A few livers were collected at week 2 post-AAV administration (A). Paraffin liver sections were prepared and immunohistochemical staining (IHC) was used to detect GFP (A, B, C, E) and CBX7 (D, F). **(A)** Almost all of hepatocytes expressed GFP at week 2 post-AAV administration. **(B)** Transposase-GFP was sparsely detected in hepatocytes at week 14 post-rAAV administration. **(C)** Control GFP and **(D)** CBX7 were expressed in nearly all of non-tumour hepatocytes at week 14 post-AAV administration. **(E)** Control GFP and **(F)** CBX7 were expressed in most, but not all, tumour cells at week 14 post-rAAV administration.

**Supplemental Figure S6**

**Fig. S6. Protein expression levels of FoxP2 in liver tumours.** Immunohistochemical (IHC) staining was conducted on paraffin sections of liver tissues harvested from mice at week 34 post-DEN injection. **(A)** Microphotos of FoxP2 IHC staining, **(B)** Quantification of FoxP2 IHC staining. Data were analysed by unpaired t test in Prism, **p<0.01. PV: portal vein; T: tumour.

**Supplemental Figure S7**

**Fig. S7. Effects of hepatic CBX7 deletion on liver cancer formation.** DEN was injected intraperitoneally once into hepatic CBX7 knockout and control WT male pups at postnatal day 12~14. Liver tissues were harvested at 34-weeks post-DEN injection. Visible tumours on the surface of each liver were measured. Graph: Scatter dot plot with bar (Mean with SEM)
